# Identification of a Novel Pleiotropic Transcriptional Regulator Involved in Sporulation and Secondary Metabolism Production in *Chaetomium globosum*

**DOI:** 10.3390/ijms232314849

**Published:** 2022-11-27

**Authors:** Shanshan Zhao, Kai Zhang, Congyu Lin, Ming Cheng, Jinzhu Song, Xin Ru, Zhengran Wang, Wan Wang, Qian Yang

**Affiliations:** School of Life Science and Technology, Harbin Institute of Technology, Harbin 150080, China

**Keywords:** pleiotropic regulator, *Chaetomium globosum*, chaetoglobosin A, sporulation, metabolome

## Abstract

Chaetoglobosin A (CheA), a well-known macrocyclic alkaloid with prominently highly antimycotic, antiparasitic, and antitumor properties, is mainly produced by *Chaetomium globosum*. However, a limited understanding of the transcriptional regulation of CheA biosynthesis has hampered its application and commercialization in agriculture and biomedicine. Here, a comprehensive study of the *CgXpp1* gene, which encodes a basic helix-loop-helix family regulator with a putative role in the regulation of fungal growth and CheA biosynthesis, was performed by employing *CgXpp1*-disruption and *CgXpp1*-complementation strategies in the biocontrol species *C. globosum*. The results suggest that the *CgXpp1* gene could be an indirect negative regulator in CheA production. Interestingly, knockout of *CgXpp1* considerably increased the transcription levels of key genes and related regulatory factors associated with the CheA biosynthetic. Disruption of *CgXpp1* led to a significant reduction in spore production and attenuation of cell development, which was consistent with metabolome analysis results. Taken together, an in-depth analysis of pleiotropic regulation influenced by transcription factors could provide insights into the unexplored metabolic mechanisms associated with primary and secondary metabolite production.

## 1. Introduction

The fungal genus *Chaetomium* isolated from different habitats is a well-known microbial cell factory that has attracted the attention of researchers because of its capacity to produce a wide array of bioactive secondary metabolites, such as terpenoids (antifungal) [1], indole alkaloid (antibacterial) [2], xanthone derivative (anti-parasitic) [3], azaphilones, and cytochalasans (antitumor) [4,5]. *C. globosum* of the genus *Chaetomium* has previously been used as a biocontrol agent owing to its capacity to produce such secondary metabolites. CheA, which is mainly produced by *C. globosum*, is the most abundant member of cytochalasans and was first reported in 1973 [6]. It has been investigated for its unique inhibitory activity against soil-borne phytopathogens, including *Sclerotinia sclerotiorum*, *Macrophomina phaseolina* [1], *Xanthomonas oryzae* [7], *Phytophthora infestans* [8], *Fusarium sporotrichioides* [9], and *Rhizopus stolonifera* [10]. Owing to its significant agricultural and commercial value, many investigators have successively analyzed this compound. The gene cluster associated with CheA biosynthesis in *Penicillium expansum* has been predicted and identified using RNA-mediated gene silencing technology [11]. Within the biosynthetic gene cluster, polyketide synthase-non-ribosomal peptide synthetase hybrids and chaperone protein enoyl reductase (ER) have been associated with the polymerization of monomeric substances to form the carbon backbone of CheA. One flavin adenine dinucleotide-dependent monooxygenase and two cytochrome P450 oxygenases, which are located downstream of the ER gene, have been used to modify a nascent substrate (prochaetoglobosin I) into a terminal product [12].

Previous research findings have revealed that the identification of novel compounds or promoting the yield of known natural products is a challenge because the majority of genes involved in secondary metabolite biosynthesis remain silent or have low expression levels under standard laboratory conditions [13,14]. A better understanding of the secondary metabolism regulation is an effective strategy for stimulating the transcription of the genes related to the biosynthetic gene cluster. Numerous studies have shown that global transcription factors, which are a class of pleiotropic regulators, are involved in the regulation of morphology, asexual development, and secondary metabolite biosynthesis in filamentous fungi [15]. One such regulator that has been extensively studied and a predominant example is *LaeA*, which was originally identified in *Aspergillus niger* and is widely distributed in fungi [16]. To date, the Zn(II)2Cys6 transcription factor *LaeA*, which functions by forming velvet complexes with *VeA* and *VelB*, has been identified as a positive regulator of the most common metabolites, including aflatoxins [17], ochratoxin A [18], mycotoxins [15], and chaetoglobosins [19]. Moreover, the overexpression of global regulators is a novel method of detecting cryptic polyketides with novel biological activities, such as antifungal and antitumor activities [20,21].

In addition, there are also many negative regulators that suppress the biosynthesis of bioactive compounds that exist in fungi. Derntl et al. described a basic helix-loop-helix (bHLH)-type regulator in *Trichoderma reesei*, which is involved in the regulation of both primary and secondary metabolisms. Inactivation of the target gene results in increased production of secondary metabolites with regard to amount and concentration; however, primary metabolism is markedly attenuated in the absence of mutants when compared to the parent cells based on phenotype and RNA sequencing analysis [22]. Furthermore, Pandit et al. observed that the deletion of the bHLH-type regulator *urdA* could remarkably enhance sterigmatocystin production in the presence of light through the activation of *aflR* expression in sterigmatocystin biosynthetic gene clusters. Deletion of *urdA* also results in substantial morphological alterations, such as conidia and cleistothecia formation even in the presence of light [23]. Over the last five years, our research group has conducted a stepwise exploration of the factors influencing CheA production. Two positive regulators, *CgLaeA* and *CgcheR*, have been detected and identified in *C. globosum* [19,24]. However, a limited understanding regarding the negative transcriptional regulation of CheA biosynthesis has hampered its application and commercialization in agriculture and biomedicine. In the present study, the *CgXpp1* gene in *C. globosum*, which encodes a bHLH-type regulator, was identified. In addition, to determine the extent of *CgXpp1* regulation, we performed a verification process based on *CgXpp1*-disruption and *CgXpp1*-complementation strategies and analyzed their effect on transcriptional regulation, CheA biosynthesis, and sporulation in *C. globosum*. Wild-type *C. globosum* W7 was used as the control. The findings of the present study could provide novel insights for improving secondary metabolite production by reducing the expression levels of negative regulators in filamentous fungi.

## 2. Results

### 2.1. Identification of the CgXpp1 Gene in C. globosum W7

According to the analysis performed using the BLASTP algorithm of NCBI (National Center for Biotechnology Information) database, CgXpp1 (GenBank: XP_001227013.1), which has a 1284-base pair (bp) open reading frame, was identified as the gene encoding the putative bHLH family of transcription regulators. The gene might be a promising switch to adjust the secondary fungal metabolism [22]. Preliminary studies of sequences revealed that the *CgXpp1* gene consists of two introns that can encode a polypeptide of 382 amino acids with a predicted molecular mass of 41.86 kDa and a theoretical isoelectric point (pI) of 7.84. The amino acid sequence of *CgXpp1* shared the highest similarity with that of *Madurella mycetomatis* (66.08%), and consisted of a highly conserved HLH-DNA binding domain with residues of 267–334 (Simple Modular Architecture Research Tool: http://smart.embl-heidelberg.de/ (accessed on 6 January 2021)) (Figure S1B). Orthologs in *CgXpp1* and other homogenous sequences were aligned using DNAMAN v6.0, and the results are presented in Figure S1A. A phylogenetic tree was generated using MEGA v6.06 and the neighbor-joining method with a bootstrap value of 1000. The results revealed that *CgXpp1* formed a distinct and stable branch with *M. mycetomatis*, which was supported by a bootstrap value of 100% in the neighbor-joining tree (Figure S1C). However, *CgXpp1* exhibited a certain level of similarity to those of other HLH proteins outside filamentous fungi, implying that *CgXpp1* possibly functions specifically as a member of the HLH family.

### 2.2. Construction of CgXpp1 Inactivation Mutants and Complementation

**Figure 1:**
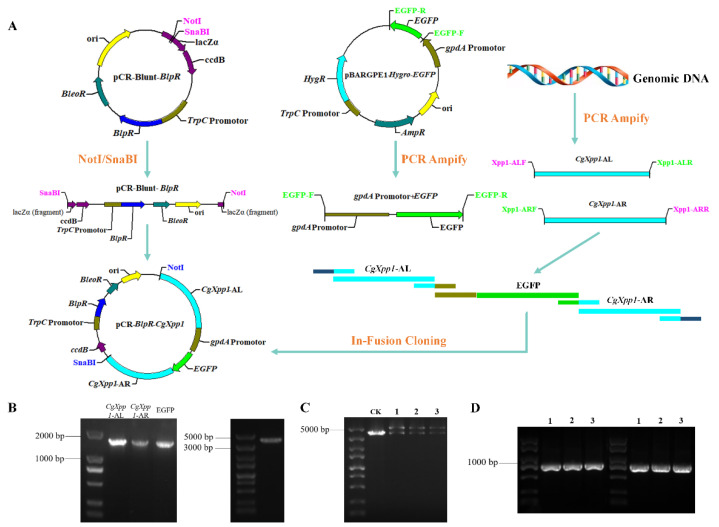
Construction schematic of the *CgXpp1* gene homologous recombination carrier and transformant verification by diagnostic PCR. (**A**) Construction diagram of the knocking-out of *CgXpp1* in *CgligD*-N3, which was derived from *C. globosum* W7. Bleomycin (*BleoR*), bialaphos (*BlpR*) resistance gene and the fluorescent protein EGFP were used as selectable markers. Regions in blue represented the homologous arms of *CgXpp1* gene. The inserted selectable marker is shown in green, and its promoter is indicated in brownish green. The *ccdB* lethal gene, which located downstream of the insertion gene, is indicated in purple; (**B**) Amplification results of the homology region *CgXpp1*-AL and *CgXpp1*-AR, as well as the selection fluorescence marker which embedded in the middle. The linearized backbone vector was obtained after double restriction digestion with NotI and SnaBI; (**C**) Double enzyme digest results of all the detected species. CK: empty control of vector pCR-Blunt-*BlpR* after NotI and SnaBI digestion. (**D**) Diagnostic PCR of all the mutants utilizing the TXF1/TXR1 and TXF2/TXR2 primer pairs.

In the present study, to examine the potential function of *CgXpp1* in CheA biosynthesis, the gene was inactivated by a homologous recombination and a flow diagram of the process is illustrated in Figure S2A and Figure 1A. A 938-bp DNA fragment (*BlpR*, Figure S2C), which replaced the *KanR* gene in the pCR-Blunt plasmid, was linked to a linearized carrier (Figure S2B) through seamless contiguity technology to produce a gene knockout skeleton vector, pCR-Blunt-*BlpR*. Due to the occurrence of the *ccdB* lethal gene on the carrier, it was chemically transformed, implemented into a survivable competent cell TransDB3.1, and selected in low-salt Luria-Bertani medium supplemented with 25 μg/mL zeocin. The transformants were further confirmed by diagnostic polymerase chain reaction (PCR) using a pair of primers, *pKan*-TF and *pKan*-TR (Table S1), which were designed based on the upstream and downstream regions of the *KanR* gene in the pCR-Blunt. According to the results, all transformants formed a fragment of 1292 bp (Figure S2D), which was verified by sequencing and was used for subsequent research. Three sequences, *CgXpp1*-AL, *CgXpp1*-AR, and selectable marker EGFP, were obtained based on the PCR amplifications (Figure 1B). Subsequently, acquired fragments were ligated into the prepared linearized carrier pCR-Blunt-*BlpR* (Figure 1B) by employing in-fusion cloning technology to generate pCR-*BlpR*-*CgXpp1*. Since the insertion sequence was not an integer multiple of 3 bp, the open reading frame of the *ccdB* gene was destroyed; therefore, subsequent construction was transformed into ordinary *Escherichia coli* DH5α-competent cells. Thereafter, we tested the mutants that had an expected infusion of the target gene as evidenced by diagnostic PCR and restriction assays. The 908-bp and 808-bp DNA fragments were accurately amplified from their derivatives using TXF1/TXR1 and TXF2/TXR2 primers (Figure 1D). Furthermore, the results of double enzyme digestion revealed that three transformants were cut into two bright bands, demonstrating that the empty vector had connected properly to the complementary sequence (Figure 1C). For the complementation construct, the unabridged open reading frame of *CgXpp1* (Figure S3A) was amplified and ligated into the linearized pCR-Blunt-*BlpR* using the same method to obtain p*BlpR*-*CgXpp1*-Com. Double enzyme digestion was performed to further confirm the complemented plasmids (Figure S3B). Two bright bands were produced by the tested strains and both sequences were 100% identical to the template. The resulting plasmids, pCR-*BlpR*-*CgXpp1* and p*BlpR*-*CgXpp1*-Com, were then introduced into the protoplasts of the *CgligD*-N3 and *CgXpp1*-inactive mutant, as described previously [25,26].

*CgligD*-N3, which lost random non-homologous recombination capacity, was identified and established in our lab to promote gene deletion in *C. globosum* W7. *CgligD*-N2–6 was obtained by dual antibiotic screening (hygromycin and bialaphos) and verified by PCR (Figure S4). Transformants could only grow on a medium containing hygromycin but not on the bialaphos-resistant plate, suggesting that the hygromycin resistance gene in the middle of the homology arm was successfully integrated into the genome and expressed appropriately (Figure S4B). In addition, two bands of 2995 bp and 2852 bp of the five mutants were obtained after PCR amplification using *CgligD*-PTF1/*CgligD*-PTR1 and *CgligD*-PTF2/*CgligD*-PTR2 primers (Figure S4C,D). However, since the gene for CK2 at the target region was *CgligD*, only one-sided primers could be integrated into the genome; therefore, there could be a non-specific amplification of the control strains. Diagnostic PCR with *CgligD*-PTF3/*CgligD*-PTR3 primers were further used for identification, a 3356-bp band was amplified from the control strain, and the size of all the five mutants was 1816 bp because of the absence of a complete *CgligD* gene in the mutants (Figure S4E). The results showed that the *CgligD* gene was completely knocked out in all the transformants; therefore, *CgligD*-N3, which further verified the correctly expressed hygromycin resistance gene by qRT-PCR (Figure S5A), was selected for the subsequent experiments.

**Figure 2:**
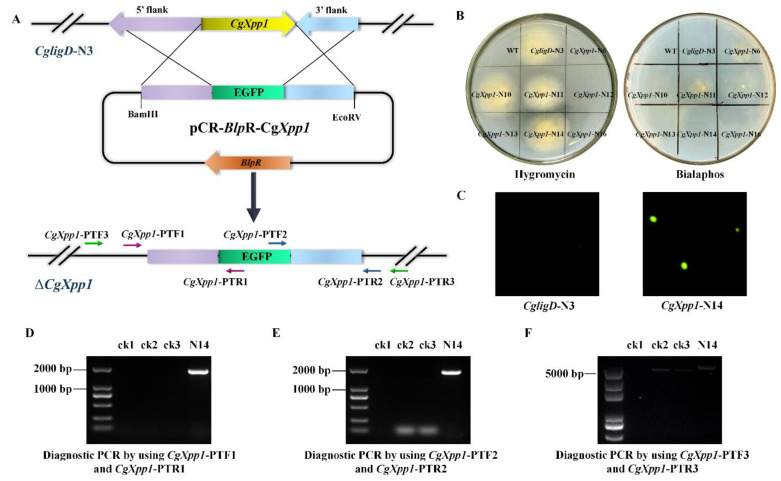
Construction and verification of the *CgXpp1* disruption mutants. (**A**) The schematic for destroying the *CgXpp1* gene in *CgligD*-N3 via the homologous recombination approach; (**B**) The double antibiotic validation consequences of *CgXpp1* genetic derivatives derived from *CgligD*-N3; (**C**) Morphological profile of the detected species and photographs of cultures observed by fluorescence microscope. (**D**–**F**) Verification of the *CgXpp1* deactivation transformants at DNA level by diagnostic PCR, which were performed with the gene-outside primers and gene-inside primers designed according to the flanking regions, respectively. ck1,the water control were used to eliminate possible pollution in the PCR reaction system; ck2, wild-type species *C. globosum* W7; ck3, *CgligD*-deleted species *CgligD*-N3; N14, the *CgXpp1* gene-destruction mutant.

The *CgXpp1* disruption mutants were initially selected on a potato dextrose agar (PDA) medium containing 80 μg/mL bialaphos. After three generations of culturing in an antibiotic-free medium, dual antibiotic screening was performed. The hygromycin resistance gene replaced the *CgligD* gene in the wild-type strain genome during the gene knockout process. Therefore, we screened the transformants that could only grow on hygromycin resistance plates. Finally, the resistant mutants were selected (Figure 2B), and the *CgXpp1* disruption derivatives were further confirmed by PCR using the primers designed based on the homologous arm of the *CgXpp1* gene and the fluorescent protein reporter gene was inserted. Two bands of 1843 bp and 1779 bp of the disruption mutant *CgXpp1*-N14 were obtained after PCR amplification using *CgXpp1*-PTF1/*CgXpp1*-PTR1 and *CgXpp1*-PTF2/*CgXpp1*-PTR2 primers (Figure 2D,E). The ck2 and ck3 did not have specific amplifications owing to the one-sided primers that were mismatched with the genomes of both strains, which distinguished them from the transformants. In addition, the gene replacement was further verified by diagnostic PCR using the *CgXpp1*-PTF3/*CgXpp1*-PTR3 primer pair, and a 5200-bp amplicon was observed in *CgXpp1*-N14. The *CgXpp1* gene was replaced by a fluorescent selectable marker *EGFP* through homologous recombination, resulting in a control strain of 4999 bp (Figure 2F), which suggests that the selectable marker cassette was integrated into the *CgXpp1* locus. In addition, green fluorescence signals (Figure 2C) corresponding to *EGFP* were coincident as inferred, confirming that the fluorescent protein gene had been integrated into the genome and correctly expressed. The relative expression level of the *gfp* gene in *CgXpp1*-N14 further provided credible evidence for this result (Figure S5B). The complement was constructed by introducing the entire *CgXpp1* gene into *CgXpp1*-N14. Similarly, the resulting mutants were confirmed by PCR (Figure S3C) and an appropriate transformant (*CgXpp1*-Com) was identified and used as a control strain for subsequent analysis.

### 2.3. Effect of CgXpp1 on C. globosum W7 Development

**Figure 3:**
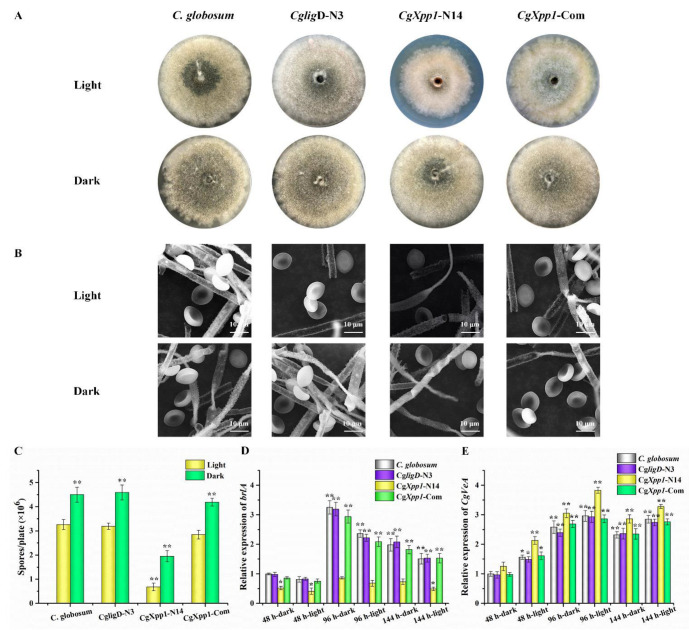
Impact of *CgXpp1* on the morphological differentiation, conidiation production and the transcription of relative genes associated with the phenotypic development when incubated under the dark and light conditions. (**A**) Sporulation of *C. globosum*, *CgligD*-N3, *CgXpp1*-N14, and *CgXpp1*-Com cultured on the PDA plates; (**B**) The morphological characteristics of the detected organisms by employing the scanning electron microscope. Bar, 10 μm; (**C**) Quantity of spores of *C. globosum*, *CgligD*-N3, *CgXpp1*-N14 and *CgXpp1*-Com that were inoculated on the PDA medium, 1 × 10^7^ spores were added in oxford cup at 28 °C under continuous light or dark conditions for 6 days. Moreover, the spores were harvested and counted using the hemocytometer; For (**D**,**E**), relative expression levels of *brlA* and *CgVeA* in *CgXpp1*-N14 and reference species grown in PDA liquid medium. The transcriptional levels of the target genes are presented relative to that of wild-type species collected after fermentation for 48 h under dark conditions, which were arbitrarily assigned a value of 1.0. The error bars represent standard deviations from three independent experiments. *p*-values were determined by Student’s *t*-test. The levels of significance were ** *p* < 0.01, * *p* < 0.05.

Generally, most regulators are essential in the regulation of secondary metabolite production and the development of filamentous fungi due to their pleiotropic effects [24,27]. To establish the function of the *CgXpp1* gene in the development of wild-type *C. globosum* W7, *CgligD*-N3, *CgXpp1*-N14, and *CgXpp1*-Com strains were cultured in a PDA medium at 28 °C under continuous light and dark conditions for 6 days. Spore production in the disruptive mutant *CgXpp1*-N14 decreased significantly, regardless of whether the culture was in the presence or absence of light when compared to the parental strain of *C. globosum* W7 and *CgligD*-N3 controls. However, sporulation was restored in the *CgXpp1*-complemented strain (*CgXpp1*-Com) (Figure 3A,C). In addition, the numbers of spores produced by *CgXpp1*-N14 strain under continuous light conditions were considerably lower than those of spores produced under dark conditions. The observation was similar to that of the control groups. The morphological characteristics of fungal strains were analyzed by culturing them in the PDA medium and observed by SEM. The aerial hyphae and substrate mycelium of all fungal strains examined were well developed and distinctly verrucose, with diameters ranging from 2.4 to 3.0 µm. The spores were olive brown, irregularly arranged, non-motile, lemon-shaped, and single-celled (8.5–9.0 × 6.9–7.3 µm); the aerial mycelium had a smooth surface (Figure 3B). No differences were observed between the reference species and the *CgXpp1*-inactive mutant. The results suggest that the *CgXpp1* gene is involved in sporulation, although it has no effect on conidial morphology and hyphal diameter. Moreover, the impacts of the *CgXpp1* gene on sporulation were not alleviated in the absence of light.

To elucidate the effect of *CgXpp1* on *C. globosum* development, the *brlA* gene, which has been identified as a critical gene for conidiophore formation in Acremonium chrysogenum [28], was examined by qRT-PCR. The expression of the *CgVeA* gene, which encodes a light-dependent regulator necessary for sporulation, was also detected in all species [29,30]. The expression of the *brlA* gene in all fungal strains tested under dark conditions was higher than that in strains incubated under light conditions (Figure 3D). In addition, the number of conidia in strains cultured under dark conditions was substantially enhanced (Figure 3A,C). In contrast, transcript levels of the *CgVeA* gene in samples grown under illumination increased considerably (Figure 3E). Similar to existing research findings [28,29], the results suggest that *brlA* could have a positive regulatory role in conidial formation, whereas *CgVeA* could have a negative role in the process. A low expression level of *brlA* was observed in the *CgXpp1*-N14 disruptive mutant relative to the controls; however, the transcript level of the *CgVeA* gene increased significantly, which could be associated with the relatively low spore production in the transformants. The results suggest that the *CgXpp1* gene could also be associated with the regulation of conidial formation.

### 2.4. Effects of CgXpp1 Disruption on CheA Production

**Figure 4:**
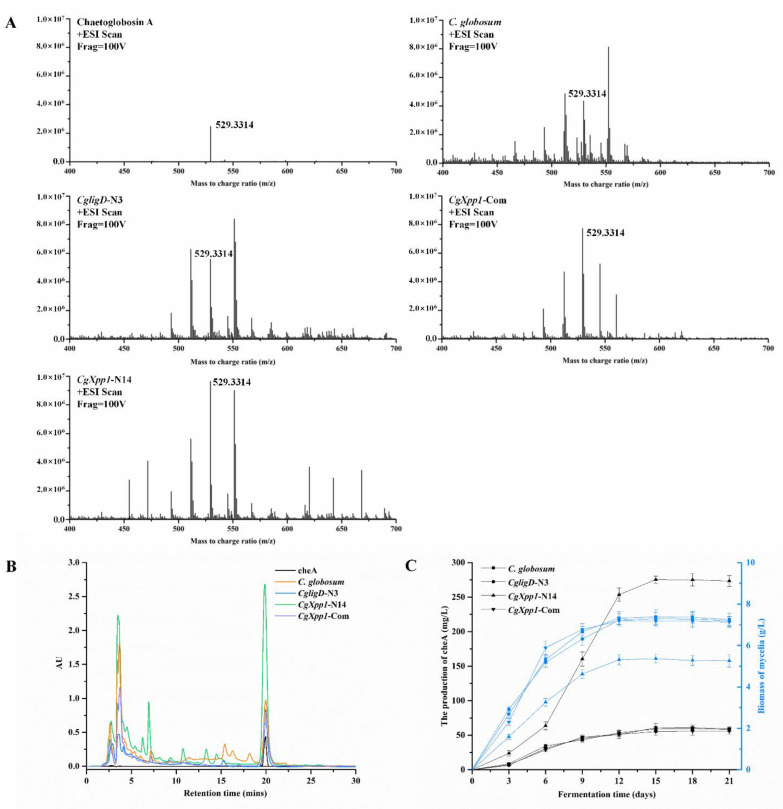
The effects of the *CgXpp1* gene on cheA production. (**A**) LC/MS analysis of the metabolites produced by *CgXpp1*-N14 and control organisms; (**B**) The yield of cheA extracted from the wild-type species and its derivatives were monitored at 227 nm by HPLC analysis; (**C**) Growth curves of isolates to be tested were obtained by cultivating in a PDA medium and sampling at three-day intervals. Biomass is expressed as dry cell weight. Quantitative cheA production of detective strains cultured in fermentation medium. Error bars show standard deviations from three independent experiments.

The production of CheA in the *C. globosum* W7, *CgligD*-N3, *CgXpp1*-N14, and *CgXpp1*-Com strains was compared, and the role of *CgXpp1* gene on its biosynthesis was investigated (Figure 4). CheA production in most inactive mutants increased to varying degrees; in particular, *CgXpp1*-N14 exhibited an increase of 339.82% relative to the controls when incubated in a PDA medium for 9 days at 28 °C (Figure 4B). Moreover, the accumulation curve of the control strains and the chromatographic peak of *CgXpp1*-N14 tended to stabilize after 15 days of incubation in PDA medium. HPLC analysis revealed that *CgXpp1*-N14 produced the highest CheA titer, which increased from 60.32 to 274.61 mg/L. However, CheA production was restored to 55.67 mg/L in the *CgXpp1*-Com strain, which was similar to that of the wild-type and *CgligD*-N3 strains (Figure 4C). The results demonstrate that the disruption of the *CgXpp1* gene in a *CgligD*-deletion background enhances CheA yield and the *CgXpp1* gene is a potential negative regulator in CheA production. To verify that the contrasting phenotypic variations observed were caused by individual nonfunctional *CgXpp1* gene fragments, the mycelial biomass of the wild-type strain, *CgligD*-N3, *CgXpp1*-N14, and *CgXpp1*-Com (complementation strain) were evaluated. A rapid increase in mycelial biomass from 3 to 12 days was observed, which reached equilibrium between 15 and 21 days. The maximum mycelial biomass of *CgXpp1*-N14 decreased considerably throughout the entire time course when compared to that of the reference strain (Figure 3C). The results suggest that *CgXpp1* exerts a negative influence on CheA production, and it is essential for cell growth and spore formation.

### 2.5. Effect of CgXpp1 on the Transcription of the CheA Biosynthetic Gene Cluster and Other Related Regulators

**Figure 5:**
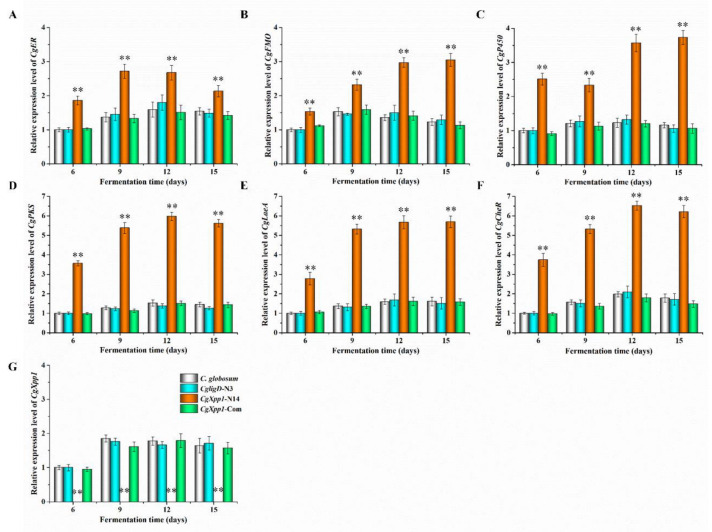
Relative expression levels of *CgER* (**A**), *CgFMO* (**B**), *CgP450* (**C**), *CgPKS* (**D**), *CgLaeA* (**E**), *CgCheR* (**F**), and *CgXpp1* (**G**) in *C. globosum* W7 (wild type), *CgligD*-N3 (*ligD* disruption mutant), *CgXpp1*-N14 (*CgXpp1* deletant) and *CgXpp1*-Com (*CgXpp1* complementary derivative) incubated in a PDA medium at various time points. The primers utilized for qRT-PCR are listed in Table S1, and the *β*-actin gene amplified from the *C. globosum* W7 genomics was used as an internal control. The transcription levels of genes involved in the target compound biosynthesis and regulation are relative to that of *C. globosum* W7 collected after fermentation for 6 days, which was arbitrarily assigned a value of 1.0. The error bars represent standard deviations from three independent experiments. *p*-values were determined by Student’s *t*-test. The levels of significance were ** *p* < 0.01.

To gain insights into how *CgXpp1* influences the fungal transcriptome, we performed qRT-PCR. *CgXpp1* deletion and reference species were grown in a PDA medium for various time points (6, 9, 12, and 15 days). The expression of four genes associated with the CheA biosynthetic gene cluster, including *CgPKS*, *CgER*, *CgP450*, and *CgFMO* were analyzed. The *CgXpp1* gene was normally expressed in the control group but not in the *CgXpp1*-N14 group (Figure 5G), demonstrating that the target fragment was successfully knocked out from the genome. In addition, the transcription profiles of *CgXpp1* in *C. globosum* W7 and *CgligD*-N3 reached the highest level at 9 days, whereas that of the *CgXpp1*-Com reached the highest level at 12 days. The gene was consistently expressed at high levels in all reference species after 9 days of incubation in a PDA medium. The high expression of *CgXpp1* could be the primary reason for repressing the constant increase in the CheA production. We observed that CheA accumulation curves for the control strains changed slowly after 9 days of incubation in PDA medium. By contrast, the CheA yield in *CgXpp1*-N14, which disrupted *CgXpp1* gene expression by homologous recombination, increased linearly from 6 to 12 days and stabilized after 15 days of incubation in a PDA medium (Figure 4C). The production of the desired compound increased significantly in *CgXpp1*-N14 when compared with that of the corresponding groups. The transcription levels of *CgER*, *CgP450*, and *CgFMO* in the *CgXpp1*-N14 strain were substantially enhanced at 12 days of incubation in a PDA medium (Figure 5A–C). Specifically, the transcription level of the *CgPKS* gene was 5.97-fold higher in the disruptive transformant than in the wild-type strain (Figure 5D). As a synthetase that influences the synthesis of the CheA backbone structure, the high expression of *CgPKS* could be associated with the relatively high titers of the final product in the *CgXpp1*-N14 mutant [12]. The findings imply that the *CgXpp1* gene could be involved in the negative regulation of the CheA biosynthetic pathway and knockout of the gene possibly activates the transcription of critical genes associated with the biosynthetic gene cluster of the target product.

Furthermore, to enhance our understanding of how *CgXpp1* functions as a regulator in *C. globosum*, the expression of regulatory genes associated with CheA biosynthesis was analyzed (Figure 5E,F). *CgLaeA* and its dynamic effects on phenotype development and secondary metabolite production have been studied extensively [16,19], as well as the transcription factor *CgcheR*, which activates the transcription of chaetoglobosin biosynthetic genes in a pathway-specific manner [24]. With regard to regulatory genes, *CgLaeA* and *CgcheR* exhibited a 5.67- and 6.52-fold increment in gene expression levels of the *CgXpp1*-N14 strain, respectively. The results suggest that the absence of the *CgXpp1* gene could effectively enhance the expression of the two regulatory factors, which could in turn, form a regulatory network with *CgLaeA* and *CgcheR* to increase production of the target compound.

To determine whether the *CgXpp1* protein plays a direct regulatory role in the biosynthesis of CheA, EMSAs were performed. The *CgXpp1* protein was assessed by SDS-PAGE and the results are displayed in Figure S6A. Furthermore, EMSAs consequences showed that *CgXpp1* has no specific bands corresponding to the probes were detected (Figure S6B). These findings illustrate that *CgXpp1* regulated the expression of the genes, which were associated with the biosynthesis gene cluster and other related regulators of CheA, in an indirect manner. Moreover, it also performed an indirect positive role in the regulation of conidial formation.

### 2.6. Analysis of the Metabolomics of C. globosum W7 and CgXpp1-N14

**Figure 6:**
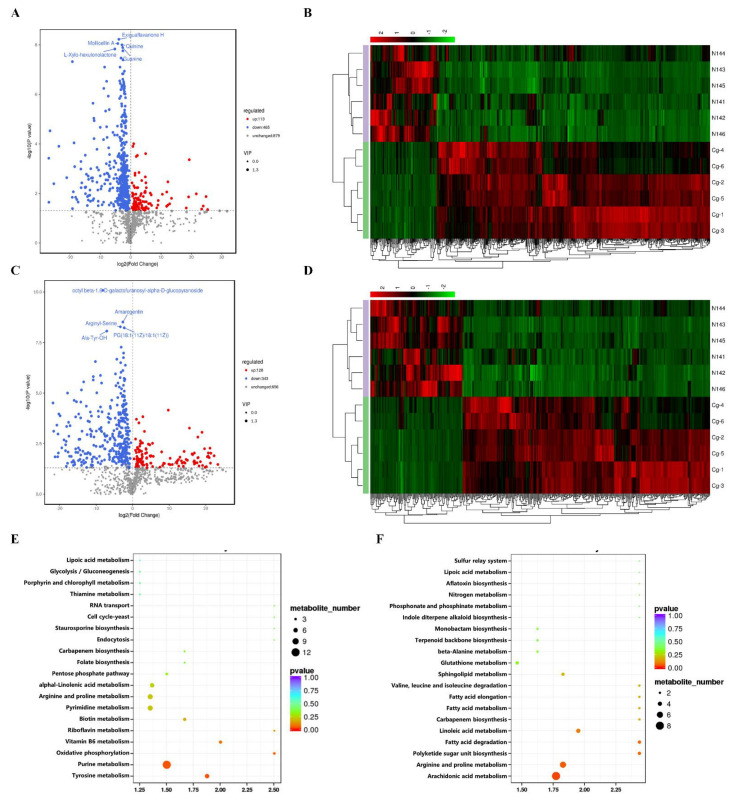
Differential metabolite analysis of the *CgXpp1*-N14 and *C. globosum* W7 species. Different metabolites volcano plot in the positive ion mode (**A**) or in the negative ion mode (**C**). Each point in the volcano plot represents a metabolite, the horizontal axis represents the multiple changes of each substance in the group compared to others (log2FoldChange), and the longitudinal axis represents the *p*-value of the *t*-test-(log10*p*-value). Markedly down-regulated metabolites are represented by blue dots, while evidently up-regulated metabolites are represented by red dots. The gray dots represent metabolites that were detected but not significantly different. The size of the dot represents the VIP value; heatmap analysis representing the differentially expressed metabolites in *C. globosum* W7 and its mutant *CgXpp1*-N14 (**B**): positive ion mode; (**D**): negative ion mode. Each horizontal column represents one sample. Kyoto Encyclopedia of Genes and Genomes (KEGG) enrichment bubble plot comparing *CgXpp1*-N14 and *C. globosum* W7 (**E**): positive ion mode; (**F**): negative ion mode. The color of the dots represents the log10*p*-value, and the redder the dot the more significant the enrichment. The size of the dots represents the number of enriched differential metabolites.

The impact of *CgXpp1* in *C. globosum* was investigated as previously mentioned analyses have focused on the influence of *CgXpp1* on spore formation, cell development and secondary metabolism production (Figure 3 and Figure 4). Therefore, metabolic profiles of the *CgXpp1* deletion strain and *C. globosum* W7 were compared by performing a metabolomics assay. Differentially expressed metabolites (DEMs) identified by liquid chromatography–tandem mass spectrometry in both positive and negative ion modes exhibited significant differences in metabolite composition between *CgXpp1*-N14 and *C. globosum* W7 (Figure 6B,D). A total of 1457 DEMs were identified in the positive ion mode, of which 578 exhibited significant differences being screened. Among them, 113 DEMs were significantly up-regulated, whereas 465 were significantly down-regulated. Furthermore, 1127 DEMs were identified in the negative ion mode, with 471 exhibiting considerable differences. Among them, 128 DEMs were significantly up-regulated, whereas 343 were significantly down-regulated (Figure 6A,C). DEMs were further subjected to the Kyoto Encyclopedia of Genes and Genomes pathway enrichment analysis. The 20 most abundant pathways included those of ‘arginine and proline metabolism’, ‘glycolysis/gluconeogenesis’, etc., which were identified in the positive ion mode, and ‘purine metabolism’ was the most significantly enriched pathway (Figure 6E). In addition, the 20 most abundant pathways identified in the negative ion mode included those of ‘fatty acid metabolism’, ‘sphingolipid metabolism’, etc. Among them, pathways associated with ‘arachidonic acid metabolism’, and ‘arginine and proline metabolism’ were the most enriched pathways (Figure 6F). The details of the DEMs obtained under the two detection modes are presented in Tables S2 and S3. Compared with the parental strain, certain numbers of primary metabolites and their related precursors exhibited downward trends when *CgXpp1* gene expression was impaired, while secondary metabolites, such as flavonoids (kanzonol W), alkaloids (isodictamnine), polyketides (manumycin A), terpenoids (ginkgolide J), and triazole drug (fluconazole) exhibited significant upward trends. The observations were consistent with those of phenotypic variation and biomass accumulation, which indicated the attenuation of the primary metabolism in the disruptive mutant of *CgXpp1*. Notably, the content of several intermediates associated with the carbon metabolism pathways, such as ‘pentose phosphate pathway’, ‘fructose and mannose metabolism pathway’, as well as ‘amino sugar and nucleotide sugar metabolism pathway’ increased significantly. The results suggest that the expression of the corresponding pathway associated with carbon metabolism was enhanced after the knockout of the *CgXpp1* gene. Abundant carbon sources could provide more precursors for the secondary metabolism, which could lead to an increase in the yield of target products.

### 2.7. Impact of the CgXpp1 Gene in the Inhibitory Activities of C. globosum W7

**Figure 7:**
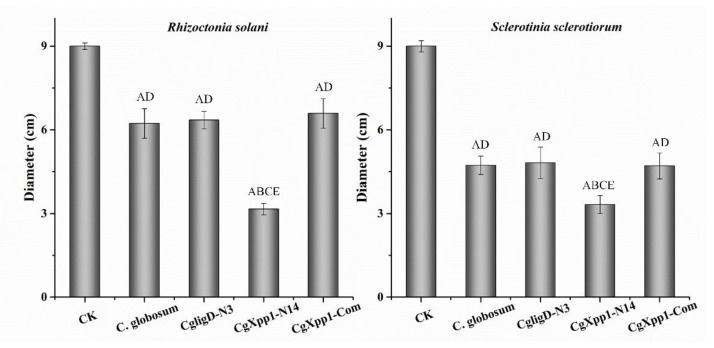
The inhibitory impacts of *CgXpp1* derivatives against phytopathogenic fungi *R. solani* and *S. sclerotiorum*. Line bars in each column denote standard errors of three repeated experiments. *p*-values were determined by Student’s *t*-test. Different lowercase letters indicate values that are significantly different (*p* < 0.05) and different majuscule letters indicate values that are significantly different (*p* < 0.01).

In the present study, culture filtrates of *C. globosum* W7, *CgligD*-N3, *CgXpp1*-N14, and *CgXpp1*-Com strains were analyzed to determine the impact of *CgXpp1* on their inhibitory activities. Diameters of colonies formed by the phytopathogenic fungi such as *Rhizoctonia solani* and *Sclerotinia sclerotiorum*, which were the well-known soil-borne pathogenic fungi, were measured (Figure 7). The colony diameters of *R. solani* and *S. sclerotiorum* on *CgXpp1*-N14 culture plates were 3.16 cm and 3.32 cm, respectively. Diameters of phytopathogenic fungal colonies that were incubated in a medium containing *C. globosum* W7 filtrate were 6.23 cm and 4.73 cm, respectively. No significant differences in antibacterial activity were observed between *CgligD*-N3 and *CgXpp1*-Com strains when compared to the wild-type strain. The results revealed that the deletion of *CgligD* had no effect on antibacterial activity, with the complement mutant of *CgXpp1* exhibiting characteristics similar to those of *C. globosum* W7. Furthermore, the diameters of phtyopathogenic fungal colonies cultured on the *C. globosum* W7 plates were larger than those of colonies cultured on *CgXpp1*-N14 plates, demonstrating that the *CgXpp1*-inactive mutant significantly increased the inhibitory activity of phytopathogenic fungi. Overall, the results indicate that *CgXpp1* markedly influences the inhibitory activity of cultured filtrates of *C. globosum*.

## 3. Discussion

Natural products isolated from the filamentous fungi, *C. globosum* are rich resources with promising biological significance. Some of the natural products, such as polysaccharides (anti-inflammatory agents) [31], cytoglobosins (antiproliferative agents) [32], 10,11-dihydroxyl-aureonitol (anticoagulant) [33], and chaetoglobosin Z (antibacterial agent) [20], have widespread use in pharmaceutical industries and as agricultural products. Cytochalasins are a typical group of secondary metabolites from *C. globosum* which have become a subject of interest among researchers owing to their structural diversity and wide range of biological activities. CheA was first reported by Seklta et al. in 1973 and was subsequently categorized as a cytochalasin with strong cytotoxicity in various tumor cells and phytopathogenic fungi [6,9,34]. Although CheA has a wide range of biological activities [7,8,9,10,35], the mechanisms associated with its regulation remain indeterminate. In addition, recent findings based on genome sequencing have demonstrated that fungi have the potential to produce more secondary metabolites than the products being explored in the laboratory [36]. Considering such drawbacks, the biosynthetic potential of natural compounds can be enhanced through regulatory mechanisms, and further research on the regulatory factors associated with the metabolic pathways of the natural products could provide a novel strategy for regulating and improving the CheA production. Over the last few years, there have been substantial advances in strategies for engineering secondary metabolite biosynthesis in fungi and drug discovery by manipulating the expression of the regulatory factors. Oakley et al. [37] identified a conserved but uncharacterized gene *mcrA* (multicluster regulator A), which functioned as a negative regulator in secondary metabolite biosynthesis in *Aspergillus nidulans* by employing a combination of genetics, molecular genetics, and genomic sequencing techniques. Knockout of *mcrA* considerably enriched the metabolic profile of the parental strain and stimulated metabolite biosynthesis in more than 10 gene clusters. In addition, knockout of the *mcrA* gene in a genetic dereplication strain in which several biosynthetic gene clusters were deleted and with the highest expression in *A. nidulans* [38] facilitated the identification of two novel compounds. *CreA*, which is a global carbon catabolite regulator, is associated with virulence and patulin biosynthesis. A previous study revealed that the loss-off-function *creA* mutants were nontoxic and lost the ability to produce patulin that was independent of *LaeA*. In the present study, analysis of sequenced *C. globosum* genomes revealed a potential bHLH-type regulator annotated as *CgXpp1*, which was located far from the CheA biosynthetic gene cluster. We first identified and demonstrated the function of the target gene using *CgXpp1*-disruption and *CgXpp1*-complementation strategies in *CgligD*-N3, which was derived from *C. globosum* W7 and the loss of random non-homologous recombination capacity.

Generally, the HLH-type regulators are closely associated with fungal morphogenesis and development, as well as the production of antibiotics from microorganisms and vegetation. In addition, some of the HLH-type regulators are crucial in plant abiotic stress regulation, drug resistance and the full virulence of the destructive pathogen [39,40]. Jin et al. identified a novel bHLH protein-encoding gene, *sclR*, which was associated with the regulation of growth and differentiation in *Aspergillus oryzae*. Exiguous sclerotia were observed on malt agar plates with inactivated mutants when compared to the parental strain; however, the number of conidia increased substantially. Moreover, numerous initial hyphae were aggregated to form more sclerotial structures in the *sclR*-overexpressing strain, although conidia production was both delayed and decreased. The results suggest that *sclR* influences the formation of asexual conidiospores and promotes sclerotial production [41]. Conversely, another bHLH-type regulator ecdR, which is crucial for early asexual development, was characterized in *A. oryzae*. Distinguished from the *sclR* gene, the *ecdR* gene disruptant resulted in a dramatically decreasing in conidia production. Moreover, with the increased expression level of *ecdR* gene, the amount of conidia generated at the early stage was enlarged [42]. Similarly with the *ecdR* gene, *CgXpp1* regulated sporulation in the strain with a *CgligD*-deletion background of the wild-type strain, and the absence of *CgXpp1* resulted in a significant decrease in conidia production even when fungal strains were cultivated in the absence of light, which is a condition conducive to spore production (Figure 3A,C). Unlike *AcstuA*, *sclR* and *aflR*, which encoded the bHLH-type and GAL4-type binuclear zinc structure regulator in filamentous fungi, impacted the morphology of hyphae and conidia, as well as influenced the formation of a stress-resistant dormant sclerotia [28,41,43]. SEM results of the present study revealed that the morphologies of spores and hyphae between the *CgXpp1* gene deletion mutants and the control strains were not significantly different (Figure 3B). Furthermore, the qRT-PCR results revealed that the expression of the *brlA* gene was significantly down-regulated at all tested time points in *CgXpp1*-N14, when compared with *C. globosum* W7 and related strains. Whereas, the transcript levels of the *CgVeA* gene were higher than those of the reference isolates (Figure 3D,E). The findings were consistent with the observed phenotypic variations. Moreover, combined with the results of EMSAs, we speculate that the *CgXpp1* gene regulates conidia formation by influencing the expression of the *brlA* and *CgVeA* genes in an indirect method.

Investigations into pleiotropic regulatory genes in microorganisms have revealed that secondary metabolite production is often closely associated with morphological development and nutrient availability. In a previous study, inactivation of the *mbsA* gene (Afu7g05620) in *Aspergillus fumigatus* caused a significant reduction in spore germination rates and chitin synthesis capacity; however, antibiotic resistance and mRNA levels of gliotoxin biosynthetic genes increased markedly [44]. Disruption of *urdA* gene expression in *A. nidulans* increased sterigmatocystin yield in a light-dependent manner, which was achieved by the up-regulation of *aflR* and *stcU* expression. Notably, some additional unknown metabolites in mutant strains lacking *urdA* have been detected by thin-layer chromatography, and the results revealed a regulatory role of the *urdA* gene (HLH-type regulator) in secondary metabolic production by filamentous fungi [23]. The bHLH transcription factors exhibit a negative regulatory effect and they can enhance regulation by triggering other transcription factors with vital regulatory functions in secondary metabolite biosynthesis, such as *anbH1*. The *anbH1* gene binds to an asymmetric E-box within the *aatA* promoter, which encodes isopenicillin N acyltransferase that catalyzes the final step of penicillin biosynthesis in *A. nidulans*. The binding site of the AnCF protein, which regulates various genes including those associated with penicillin biosynthesis, such as *ipnA* and *aatA*, is located in the *aatA* promoter region [45,46]. Consistent with our findings, *CgXpp1* is a regulator that not only promotes conidia formation but also indirect negatively regulates CheA biosynthesis (Figure 4). In addition, knockout of the target gene increased the transcription levels of key genes associated with the CheA biosynthetic gene cluster, as well as other regulators (*CgLaeA* and *CgcheR*) involved in the biosynthetic pathway (Figure 5). *CgLaeA* and *CgcheR* are essential for antibiotic production and the deletion of either of the genes leads to impaired or reduced production of the target compounds [19,24]. The high transcription levels of *CgLaeA* and *CgcheR* might be the indirect reason that explains the remarkable enhancement of the cheA yield observed in the present study by the absence of the *CgXpp1*. Activation of the expression of natural product biosynthetic gene clusters often involves complex regulatory networks, with the phenomenon of interactions among multiple regulators existing in various microorganisms, including the *SbbR*/*SbbA* (ArpA/AfsA-type) regulatory system and the *LaeA*/*VeA*/*VelB* velvet complexes [15,18,47].

Furthermore, the HLH family of regulators not only links the primary and secondary metabolism but also influences the polysaccharide metabolism [22,48]. The *AodevR* gene, which significantly influences the chitin and starch metabolism, has been identified and characterized in *A. oryzae*. Notably, the overexpression of the *AodevR* mutant effectively inhibits the transcription of amylase activity-related genes that are associated with starch degradation. Comparative proteomic analysis was employed to explore the functions of the *sclR* gene, and the results revealed that the multifunctional regulator is significantly associated with the regulation of carbon metabolism, secondary metabolite biosynthesis, and carbohydrate metabolism in *A. oryzae*. Differentially expressed proteins and API-ZYM analysis revealed that α-amylase (AO090023000944; EC 3.2.1.1), which is an essential enzyme that hydrolyses the α-linked polysaccharides, was markedly upregulated in the *sclR*-destructive mutant. Efficient hydrolysis of starch and glycogen could produce large amounts of glucose and maltose for industrial production [49]. A similar phenomenon was observed in the present study. The deletion of the *CgXpp1* gene had a significant effect on multiple metabolisms of the target species, including the ‘fatty acid metabolism’, ‘purine metabolism’, and ‘arginine and proline metabolism’. The restriction of amino acid and fatty acid metabolisms may contribute to the abnormal morphology of the *CgXpp1* disruption species cultured in a PDA liquid medium. In addition, metabolome analysis also revealed that the contents of several intermediates involved in carbon metabolism pathways increased considerably, suggesting that the knockout of the *CgXpp1* gene could stimulate the expression of corresponding carbon metabolism pathways when compared with the wild-type strain (Tables S2 and S3). An increase in carbon sources could lead to a significant increase in the quantity of substrates required for secondary metabolite production, which could in turn, increase target product yields.

In summary, the present study identified a novel bHLH-type pleiotropic regulator *CgXpp1*, which had a positive effect on spore formation and a negative effect on CheA production. The results of the present study provide novel insights into the metabolic regulatory mechanisms associated with primary and secondary metabolism in *C. globosum*. However, further studies investigating the regulatory targets of the HLH family of regulators in other genera of filamentous fungi are required to gain a comprehensive understanding of their roles in metabolic networks.

## 4. Materials and Methods

### 4.1. Strains, Plasmids, Primers, Culture Media, and Growth Conditions

*CgligD*-N3, a *ligD*-deficient species incapable of random non-homologous recombination, is derived from *C. globosum* W7, (CGMCC 3.14974) which was obtained from the Microbial Genetic Engineering Lab of Harbin Institute of Technology. Other strains and plasmids used in this study are summarized in Table 1; primers are listed in Table S1. The *E. coli* strains DH5α and TransDB3.1 cells, grown in Luria-Bertani and low salinity Luria-Bertani medium supplemented with appropriate concentrations of antibiotics, were employed for cloning purposes. *C. globosum* W7 (the control isolate) and *CgligD*-N3 and its derivatives were cultivated at 28 °C statically on potato dextrose agar (PDA), which was composed of potato 200 g, dextrose 20 g, agar 15 g, and 1000 mL water, for spore preparation and morphological comparison. When required, experiments in Petri dishes were made with the same media supplemented with hygromycin (200 ug/mL) or bialaphos (80 ug/mL) for screening the gene disruption mutants and the complemented strains. Moreover, for CheA production and the biomass analysis, the prepared spore suspensions of isolates *CgligD*-N3 and its mutants were inoculated into 50 mL PDA broth in 150 mL flasks at 180 rpm, sampled, and tested at various time points. The media used for the transformation of protoplasts of *CgligD*-N3 were the same as previously reported [50].

### 4.2. In Silico Analysis of CgXpp1 and Construction of the Phylogenetic Tree

The amino acid sequence of *CgXpp1* (GenBank: XP 001227013.1) was utilized as the query for a BlastP algorithm analysis at the online platform NCBI (National Center for Biotechnology Information). In addition, its orthologous proteins were aligned by using multiple protein alignment tool DNAMAN 6.0 software, and the phylogenetic tree was reconstructed with the neighbor-joining algorithms by employing Molecular Evolutionary Genetics Analysis (MEGA) software version 6.06 to further determine homology [51,52]. The phylogenetic distance matrices were estimated by the Jones–Taylor–Thornton (JTT) model [53]. The stability of clades in the trees was evaluated by bootstrap analysis based on 1000 replicates [54].

### 4.3. Construction of CgXpp1-Disruption Mutants and Their Complementation

The *CgXpp1* gene disruption was performed by the strategy of homologous recombination. To construct the *CgXpp1*-disruption mutant, a 1.5 kb DNA fragment corresponding to the region upstream of *CgXpp1* (*CgXpp1*-AL) and a 1.5 kb DNA fragment corresponding to the region downstream of *CgXpp1* (*CgXpp1*-AR) were amplified with the *C. globosum* W7 genomic DNA as template by polymerase chain reaction (PCR) using the *CgXpp1*-ALF/*CgXpp1*-ALR and *CgXpp1*-ARF/*CgXpp1*-ARR primer pairs (Table S1), respectively. Bialaphos resistance gene (*BlpR*) and green fluorescent protein EGFP selectable marker, which were obtained from the plasmids pBARGPE1-mCherry and pBARGPE1-*EGFP*, were amplified by PCR using as primers oligonucleotides *BlpR*-F/*BlpR*-R and *EGFP*-F/*EGFP*-R (Table S1). A 795 bp fragment of the kanamycin resistance gene in pCR-Blunt was replaced by *BlpR* to generate the skeleton vector pCR-Blunt-*BlpR* for gene knockout, and the construction process was as follows. The linearization vector was obtained by PCR amplification with the primer pair pBlunt-F/pBlunt-R. Whereafter, the linearized carrier and the *BlpR* fragment, which contained 20–25 bp homologous regions on both sides of the segments, were connected as per the instructions provided on the ClonExpress MultiS One Step Cloning Kit (Vazyme). They were then chemically transformed into *Escherichia coli TransDB3.1* competent cell, and the transformants were verified by agarose gel electrophoresis. These three sequences (*CgXpp1*-AL, *EGFP* and *CgXpp1*-AR) were ligated into pCR-Blunt-*BlpR*, which was prepared by digesting with the restriction enzymes NotI and SnaBI and the similarity method mentioned above was utilized to generate pCR-*BlpR*-*CgXpp1*.

Complementation was performed with pCR-Blunt-*BlpR* carrying *CgXpp1*. The primers *CgXpp1*-ComF/*CgXpp1*-ComR (Table S1) were employed to amplify the 1284 bp DNA fragment containing the *CgXpp1* from genomic DNA of *C. globosum* W7. Meanwhile, pCR-Blunt-*BlpR* was digested with EcoRV and NotI, and subsequently the DNA fragment of *CgXpp1* was fused with the linearizing vector to generate p*BlpR*-*CgXpp1*-Com.

### 4.4. Transformation of C. globosum W7 Protoplasts

After constructing the plasmids pCR-*BlpR*-*CgXpp1* and p*BlpR*-*CgXpp1*-Com, PEG4000-mediated plastid transformation was performed to acquire the inactivating and complementary strains. The resulting plasmid was introduced into the protoplasts of the *CgligD*-N3 and *CgXpp1*-inactive mutants, as described previously [25,26]. Transformational strains were selected by their resistance to hygromycin (200 μg/mL) and bialaphos (80 μg/mL). Following screening and purification, all genetic modification products were maintained on the PAD medium for spore collection and preserved as glycerol suspensions (50%, *v/v*) at −80 °C.

### 4.5. Morphological Studies

To evaluate the effect of *CgXpp1* on *C. globosum* development, morphology properties of the *C. globosum* W7, *CgligD*-N3, *CgXpp1*-inactive, and *CgXpp1*-complementary derivatives were observed by light microscopy (Nikon ECLIPSE E200) and scanning electron microscopy (Cambridge S-250). All tested species were inoculated in oxford cups (each oxford cup was inoculated with the same concentration spores), on a PDA medium at 28 °C under continuous light or dark conditions for 6 days, and the colony morphologies were compared. Experiments were performed in triplicate. Samples for scanning electron microscopy (SEM) were prepared by the method described by Guan et al. [55]. Firstly, a square was cut from an agar plate and then fixed at 2.5% glutaraldehyde buffer (pH 7.2) at 4 °C for approximately 1.5 h. Subsequently, after washing twice with phosphate buffer, all the tested specimens were dehydrated with a graded series of ethanol from 50% to 100%. All samples were then passed through tert-butanol and critical-point drying by the lyophilizer. Lastly, the dried samples were mounted onto a stub-bearing adhesive, sputter-coated with a gold layer, and viewed in a Cambridge S-250 scanning electron microscope.

### 4.6. Determination of Mycelial Biomass and CheA

For the determination of the mycelial biomass, fifty-milliliter cell cultures grown in the PDA liquid medium were washed three times by deionized water, collected by vacuum filtration, and dried at 60 °C to a constant weight to determine the dry cell weight. CheA production was assayed from detective species in a liquid PDA medium inoculated with approximately 1 × 10^7^ spores at 28 °C. Samples were first centrifuged at 12,000 rpm for 10 min to remove the cells, and then extracted with equal volumes of ethyl acetate (EtOAc) which involved shacking treatment overnight. The samples were dehydrated with anhydrous sodium sulfate and enriched in a rotary evaporator at 35 °C. After that, the crude extract was dissolved in 1.5 mL of MeOH and filtered using the 0.22 mm polyethylene filter before injection. Subsequently, the extractives of all determining samples were then subjected to reversed-phase HPLC analysis using a Waters 2695–2489 system equipped with a TC-C18 column (Agilent, 4.6 mm × 250 mm, 5 um) on an isocratic elution condition of 45% CH_3_CN (*v/v*) in H_2_O at a flow rate of 1.0 mL/min, and the detection wavelength was 227 nm [12]. The fermentation cultures were also measured by an Agilent 6520 Accurate-Mass QTOF LC/MS system (Agilent Technologies, Palo Alto, CA, USA) equipped with an electrospray ionization source under the same condition. CheA (C_32_H_36_N_2_O_5_) standard used as the control was purchased from Sigma-Aldrich (Germany). These experiments were all performed in triplicate.

### 4.7. RNA Isolation and Quantitative Real-Time PCR

Transcription levels of *CgXpp1* and the relative genes of cheA biosynthetic gene clusters were compared between *C. globosum* W7, *CgligD*-N3, *CgXpp1*-inactive, and *CgXpp1*-complementary mutants by quantitative real-time PCR (qRT-PCR) analysis. For that, total RNA from *C. globosum* W7, *CgligD*-N3 and its mutants was isolated from mycelia grown at 28 °C at various time points (6, 9, 12, and 15 days) in a liquid PDA medium. The detailed steps for RNA extraction, quality examination, and quantification were described previously by Long et al. and Zhang et al. [56,57]. The RT-qPCR experiments were implemented on ABI 7500 (Applied Biosystems) using Go Taq^®^ qPCR Master Mix (Promega). The running conditions were the same as mentioned formerly by Liu et al. [50]. The sequences of the gene-specific primers used in qRT-PCR were developed with Primer Premier 5.0 and are listed in Table S1. The *β*-actin gene (accession number: CH408033.1) was used as an internal control which was obtained from the genomics of *C. globosum* W7. The relative gene expression levels (2^−ΔΔCT^) were measured automatically [58]. Results were conducted in triplicate and each experiment was also repeated three times.

### 4.8. Metabolome Analysis

The wild-type species *C. globosum* W7 and *CgXpp1*-nonfunctional derivative were singly cultured in 50 mL of sterile PDA broth and fermented at 180 rpm and 28 °C for 7 days, respectively. The cells were then harvested by centrifugation at 5000 rpm for 10 min, and the supernatant was discarded. Subsequently, the cells were washed with sterilized water until the solvent of the extracellular substances did not appear to remove any more colored material. These samples were stored in liquid nitrogen and metabolome data statistics were performed on a platform (Biomarker Technologies Corporation, Beijing, China). The LC/MS system for metabolomics analysis is composed of Waters Acquity I-Class PLUS ultra-high performance liquid tandem Waters Xevo G2-XS QT of high-resolution mass spectrometer. The column used was purchased from Waters Acquity UPLC HSS T3 column (2.1 mm × 100 mm, 1.8 μm). The eluents for the positive polarity mode and negative polarity mode were eluent A (0.1% formic acid aqueous solution) and eluent B (0.1% formic acid acetonitrile). The identified compounds were searched for classification and pathway information in the KEGG, HMDB, and lipidmaps databases. According to the grouping information, the difference multiples were calculated and compared and the T test was used to calculate the different significance p values of each compound. The method of combining the difference multiple, the *p*-value, and the VIP value of the OPLS-DA model was adopted to screen the differential metabolites. The screening criteria were Fold change > *p*-value < 0.05 and VIP > 1. The difference metabolites of KEGG pathway enrichment significance were calculated using the hypergeometric distribution test.

### 4.9. Determination of the Antifungal Activity of Liquid Filtrates against Phytopathogenic Fungi

The concentrated culture filtrates of *C. globosum* W7, *CgligD*-N3, *CgXpp1*-inactive and *CgXpp1*-Com mutants were incorporated into a PDA medium to achieve a final concentration of 1% (*v/v*), and the PDA medium without the extracts was used as the negative control. Single inoculated chips with phytopathogenic fungi from 3-day-old PDA cultures were inoculated on the center of PDA mixture plates. When the negative control fungi spread over the whole plates, the colony diameters of the different treatments were measured by the vernier caliper. All experiments were performed in triplicate.

### 4.10. Expression and Purification of the CgXpp1 Protein

The codon-optimized *CgXpp1* gene, which encoded a HLH type regulator, was amplified by employing *CgXpp1*-CPF and *CgXpp1*-CPR primer pairs. After digesting the pET-28a plasmid with XhoI and MluI, the obtained fragment was fused to the linearized vector by seamless splicing to generate pET-28a-*CgXpp1*. Subsequently, pET-28a-*CgXpp1* was chemically transformed into *E. coli* BL21 (DE3) cells for heterologous expression, and then the validated transformers were incubated in an LB liquid medium in a shaking incubator at 180 rpm and 37 °C. The *CgXpp1*-His6 was induced by adding isopropyl thio-D-galactopyranoside (0.5 mM) to the culture and cultivating it at 15 °C for 28 h The culture was collected and mixed with 1 × PBS. After ultrasonication, the supernatant was collected, which was the *CgXpp1* protein. The nickelnitrilotriacetic acid (Ni-NTA) agarose chromatography was used to purify the *CgXpp1* based on the protocol of the manufacturer. In addition, the purified protein was evaluated by SDS-PAGE and the concentration was measured by a BCA protein assay Kit (Novagen). Finally, the protein samples were stored in 5% glycerol at −80 °C or directly used in the experimental test.

### 4.11. Electrophoretic Mobility Shift Assays (EMSAs)

Electrophoretic mobility shift assays were carried out as previously described by Wang et al. and Li et al. [29,59]. The DNA probes containing the promoter regions (500-bp: from −400 to +100) of *CgPKS*, *CgP450*, *CgFMO*, *CgER*, *brlA*, *CgVeA*, *CgLaeA*, and *CgcheR*, were PCR-amplified using the primers presented in Table S1. Then, the obtained DNA probe (10 ng) was incubated with various concentrations of *CgXpp1* protein (0–1.6 µg with intervals of 0.4 µg) in a 20 µL reaction mixture, which was comprised of 20 mM Tris base, 2 mM dithiothreitol (DTT), 5 mM MgCl_2_, 0.5 µg calf BSA, and 5% (*v/v*) glycerol. The free DNA and DNA–protein complexes were separated by electrophoresis on non-denaturing 4% (wt/vol) polyacrylamide gel for 25 min, with 0.5 × TBE as the running buffer. Subsequently, the DNA was stained with SYBR Gold (1 µL) for 30 min and photographed under UV by utilizing Quantity One.

### 4.12. Statistical Analysis

In order to determine the significance of treatment differences, all experiments from three parallel measurements were measured independently, and the mean values ± SD were presented. Student’s *t*-test was used to analyze the data. *p*-value was used as a standard criterion of statistical significance. * *p* < 0.05, ** *p* < 0.01.

## Figures and Tables

**Table 1:** Strains and plasmids employed in this study.

Strain or Plasmid	Description	Source or Reference
*C. globosum* W7	Parental strain; chaetoglobosin A producer	This study
*CgligD*-N3	*CgligD* deletion mutant	This study
*CgXpp1*-N14	*CgXpp1*-deletion mutant	This study
*CgXpp1*-Com	*CgXpp1*-complemented mutant	This study
		
DH5α	Host strain for cloning	Vazyme Biotech Co., Ltd, Nanjing, China
*Trans*DB3.1	*ccd*B gene survival competent cell, used to propagate and maintain vectors containing the *ccd*B gene	TransGen Biotech, Beijing, China
*E. coli* BL21 (DE3)	Heterologous expression host	Comate Biosciences Co., Ltd, Changchun, China
		
pCR-Blunt	Routine DNA cloning contains bleomycin (*BleoR*) and kanamycin (*KanR*) resistance gene, the vector involvement of a *ccdB* lethality gene	Thermo Fisher Scientific, Carlsbad, CA, USA
pET-28a	Prokaryotic expression vector	Comate Biosciences Co., Ltd, Changchun, China
pBARGPE1-mCherry	Obtaining bialaphos resistance gene (*BlpR*)	Miaoling company, Wuhan, China
pCR-Blunt-*BlpR*	Skeleton vector for gene knockout, contains bleomycin (*BleoR*) and bialaphos (*BlpR*) resistance gene based on pCR-Blunt	This study
pBARGPE1-*EGFP*	Obtaining green fluorescent protein EGFP selectable marker	Miaoling company, Wuhan, China
pET-28a-*CgXpp1*	Heterologous expression vector of *CgXpp1* gene	This study
pCR-*BlpR*-*CgXpp1*	*CgXpp1-*deletion vector based on pCR-Blunt-*BlpR*	This study
pBlpR-*CgXpp1*-Com	*CgXpp1*-complemented vector based on pCR-Blunt-*BlpR*	This study

## Data Availability

All data are contained within this manuscript.

## Supplementary Materials

The following supporting information can be downloaded at: https://www.mdpi.com/article/10.3390/ijms232314849/s1. Figure S1. Bioinformatic analysis of CgXpp1 in *C. globosum* W7. (A) Multiple Sequence Alignment of CgXpp1 orthologs from similarity species, which obtained from the GenBank databases, performed by using DNAMAN software. The predicted putative nuclear localization signal (NLS) and helix-loop-helix (HLH) domains are presented by green and blue lines respectively; (B) The structure of CgXpp1 regulator on evaluated by the online Simple Modular Architecture Research Tool (SMART) (available online: http://smart.embl-heidelberg.de/). Bright purple rectangle: regions of low compositional complexity; HLH region: helix-loop-helix (HLH) domain; (C) Neighbor-joining tree showing the phylogenetic position of isolate *C. globosum* W7 and its homologs based on amino acid sequences. CgXpp1 of *C. globosum* W7 is highlighted in bold. The Clustal X 1.83 program was used for performing sequence alignment. Only bootstrap values above 50 % (percentages of 1000 replications) are indicated. Bar, 0.05 amino acid substitutions per site. Figure S2. Schematic for gene knockout backbone vector pCR-Blunt-*BlpR*. (A) Construction diagram of carrier pCR-Blunt-*BlpR*. Bleomycin (*BleoR*), bialaphos (*BlpR*) resistance gene and a *ccdB* lethality gene were employed as the selectable marker; (B) The linearized carrier of pCR-Blunt obtained by PCR amplifying; (C) Bialaphos resistance gene (*BlpR*), which was employed to replace the *KanR* gene in the pCR-Blunt plasmid, was amplified from the vector pBARGPE1-mCherry using the *BlpR*-F and *BlpR*-R primers pairs; (D) Diagnostic PCR of all the mutants. 1-4: represented different transformants. Figure S3. Construction the complementation plasmid of *CgXpp1*. (A) The amplified result of the unbroken *CgXpp1* by using as primers oligonucleotides *CgXpp1*-ComF and *CgXpp1*-ComF; (B) *CgXpp1*-Com double enzyme digestion verification. CK: pCR-Blunt-*BlpR* digested with EcoRV and NotI. 1-4: represented various mutants that formed a linearized fragment of empty carrier (3639 bp) and an inserted sequence (1290 bp) after digesting by the same restriction endonuclease; (C) Diagnostic PCR of all the *CgXpp1* complementation mutants. CK: Original species *CgXpp1*-N14, which has been verified to completely knockout the target gene. Figure S4. Construction and validation of the *CgligD* disruption mutants. (A) The strategy for knocking-out the *CgligD* gene in the wild-type species via homologous recombination method; (B) The double antibiotic verification results of *CgligD* genetic derivatives derived from *C. globosum* W7; (C–E) Verification the *CgligD* disruption mutants at DNA level by diagnostic PCR. ck1, water control; ck2, wild-type strain *C. globosum* W7; N2-N6, the *CgligD* gene deletant named *CgligD*-N2–*CgligD*-N6. Figure S5. Relative expressions of hygromycin resistance gene (*hygR*) and green fluorescent reporter gene (*gfp*) in GFP-CK (parental strain carrying empty vector pBARGPE1-*EGFP*), *C. globosum*, *CgligD*-N3 (*CgligD* deletion mutant), *CgXpp1*-N14 (*CgXpp1* disruption mutant) and *CgXpp1*-Com (*CgXpp1* complemented derivative) at 9 days of incubation in PDA medium at 28 °C. Data were averaged using triplicate measurements. Figure S6. EMSAs for detecting the binding ability of purified CgXpp1 protein to the promoter regions of *CgPKS*, *CgER*, *CgP450*, *CgFMO*, *CgcheR*, *CgLaeA*, *CgVeA* and *brlA*. (A) Validation of the purified CgXpp1 protein by SDS-PAGE; (B) EMSA of the CgXpp1 protein and detected gene probes. Lanes 1–5: 0, 0.4, 0.8, 1.2 and 1.6 µg of CgXpp1, respectively. Table S1. Primers designed for *C. globosum* W7 in this work. Table S2. Metabolites identified that were differentially abundant between *C. globosum* W7 and *CgXpp1*-N14 in the positive ion mode, based on fold *P* < 0.05. **Table S3**. Metabolites identified that were differentially abundant between *C. globosum* W7 and *CgXpp1*-N14 in the negative ion mode, based on fold *P* < 0.05.
