# Supplementary material for: Identification of a Novel Pleiotropic Transcriptional Regulator Involved in Sporulation and Secondary Metabolism Production in Chaetomium globosum
Source: Int J Mol Sci. 2022 Nov 27;23(23):14849. doi: 10.3390/ijms232314849 (PMC9740612; doi:10.3390/ijms232314849)
Supplement: Supplementary file 1 [file ijms-23-14849-s001.zip › ijms-1961454-supplementary.pdf]

Identification of a novel pleiotropic transcriptional regulator involved in sporulation and secondary metabolism production in *Chaetomium globosum*

Shanshan Zhao, Kai Zhang, Congyu Lin, Ming Cheng, Jinzhu, Song, Xin Ru , Zhengran Wang, Wan Wang, Qian Yang*

School of Life Science and Technology, Harbin Institute of Technology, Harbin 150080, China

* **Correspondence**: yangq@hit.edu.cn; Tel./fax: +86-451-86402652

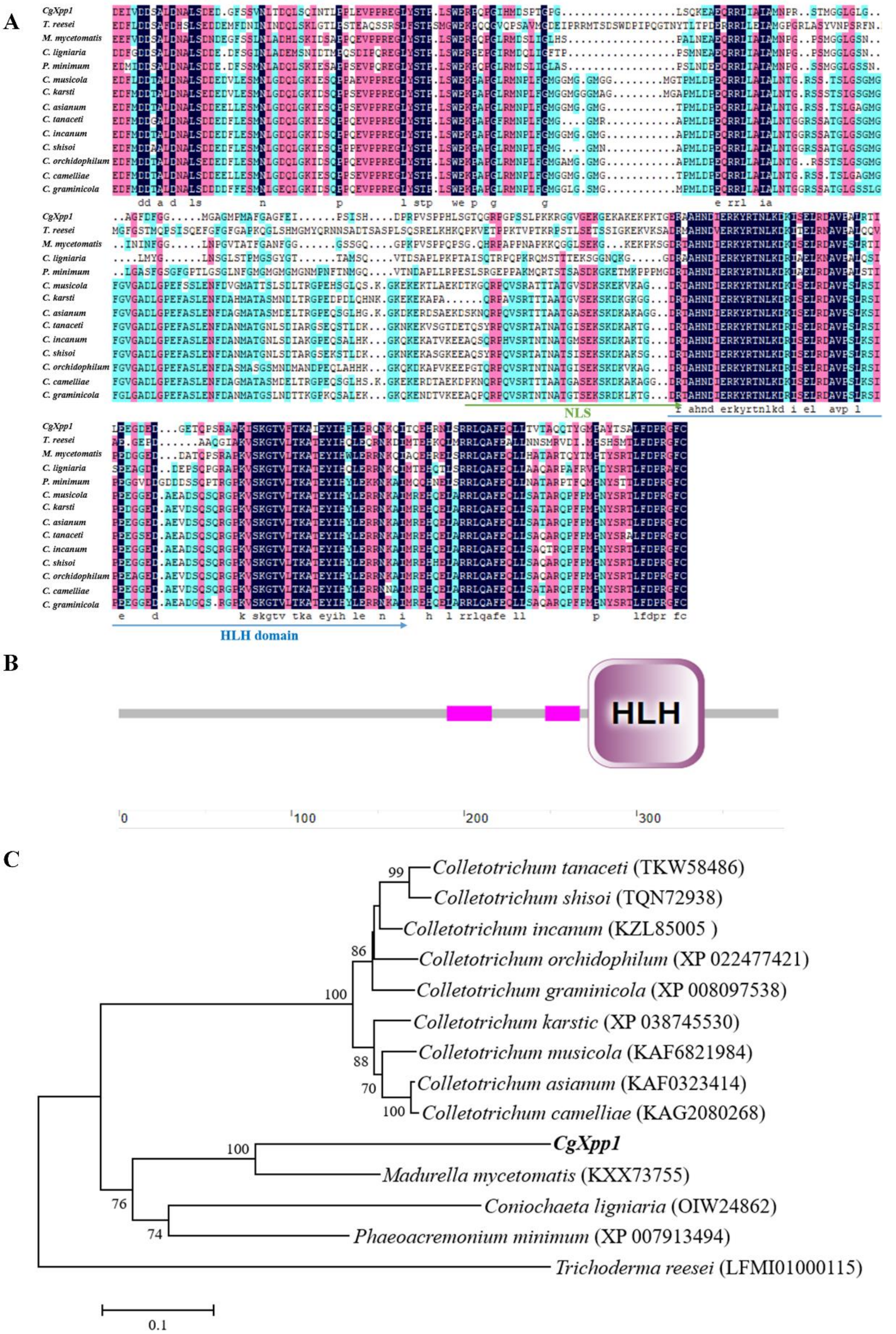

Figure S1. Bioinformatic analysis of CgXpp1 in *C. globosum* W7. (A) Multiple Sequence Alignment of CgXpp1 orthologs from similarity species, which obtained from the GenBank databases, performed by using DNAMAN software. The predicted putative nuclear localization

signal (NLS) and helix-loop-helix (HLH) domains are presented by green and blue lines respectively; (B) The structure of CgXpp1 regulator on evaluated by the online Simple Modular Architecture Research Tool (SMART) (available online: http://smart.embl-heidelberg.de/). Bright purple rectangle: regions of low compositional complexity; HLH region: helix-loop-helix (HLH) domain; (C) Neighbor-joining tree showing the phylogenetic position of isolate *C. globosum* W7 and its homologs based on amino acid sequences. CgXpp1 of *C. globosum* W7 is highlighted in bold. The Clustal X 1.83 program was used for performing sequence alignment. Only bootstrap values above 50 % (percentages of 1000 replications) are indicated. Bar, 0.1 amino acid substitutions per site.

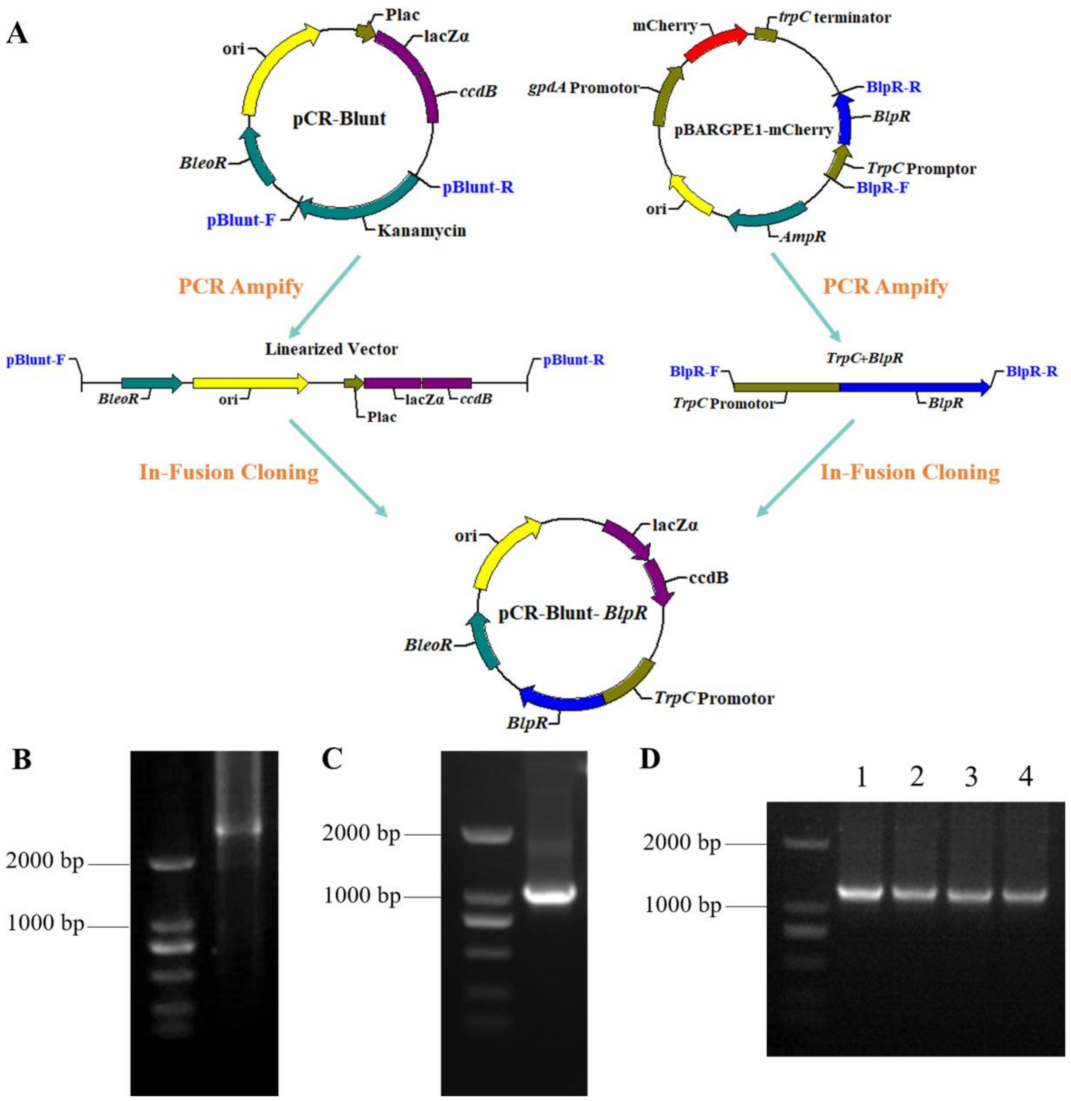

Figure S2. Schematic for gene knockout backbone vector pCR-Blunt-*BlpR*. (A) Construction diagram of carrier pCR-Blunt-*BlpR*. Bleomycin (*BleoR*), bialaphos (*BlpR*) resistance gene and a *ccdB* lethality gene were employed as the selectable marker; (B) The linearized carrier of pCR-Blunt obtained by PCR amplifying; (C) Bialaphos resistance gene (*BlpR*), which was employed to replace the *KanR* gene in the pCR-Blunt plasmid, was amplified from the vector pBARGPE1-mCherry using the *BlpR*-F and *BlpR*-R primers pairs; (D) Diagnostic PCR of all the mutants. 1-4: represented different transformants.

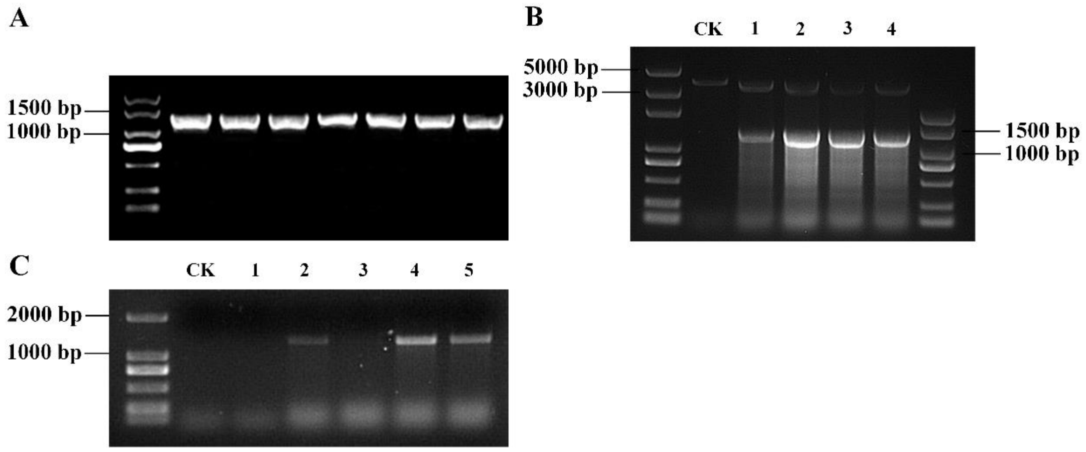

Figure S3. Construction the complementation plasmid of *CgXpp1*. (A) The amplified result of the unbroken *CgXpp1* by using as primers oligonucleotides *CgXpp1*-ComF and *CgXpp1*-ComF; (B) *CgXpp1*-Com double enzyme digestion verification. CK: pCR-Blunt-*BlpR* digested with EcoRV and NotI. 1-4: represented various mutants that formed a linearized fragment of empty carrier (3639 bp) and an inserted sequence (1290 bp) after digesting by the same restriction endonuclease; (C) Diagnostic PCR of all the *CgXpp1* complementation mutants. CK: Original species *CgXpp1*-N14, which has been verified to completely knockout the target gene.

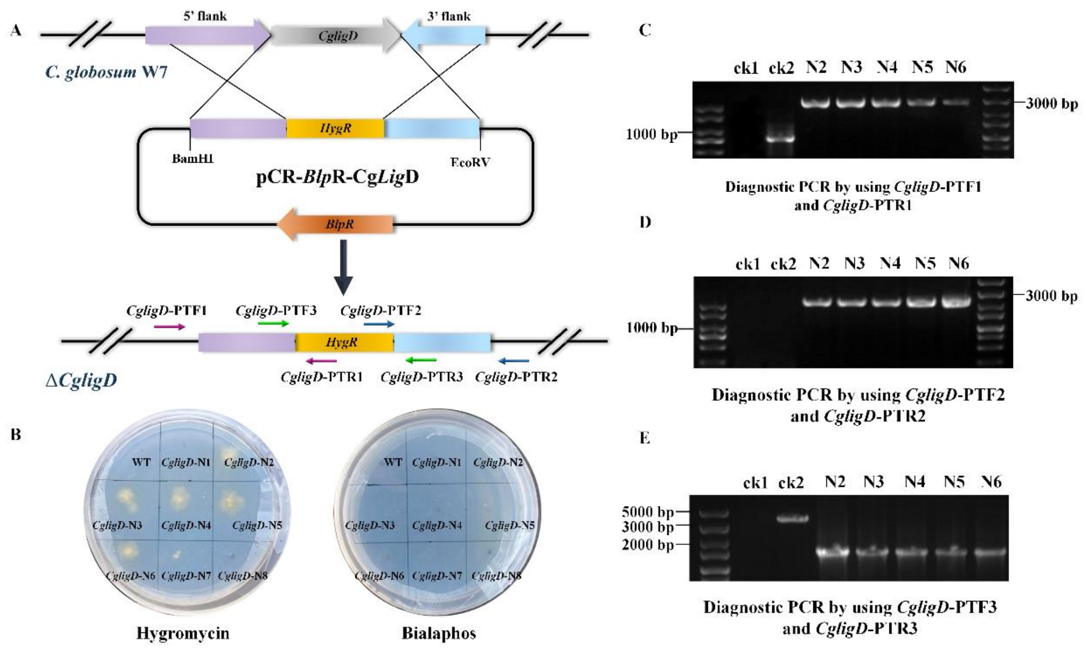

Figure S4. Construction and validation of the *CgligD* disruption mutants. (**A**) The strategy for knocking-out the *CgligD* gene in the wild-type species via homologous recombination method; (**B**) The double antibiotic verification results of *CgligD* genetic derivatives derived from *C. globosum* W7; (**C**)-(**E**) Verification the *CgligD* disruption mutants at DNA level by diagnostic PCR. ck1, water control; ck2, wild-type strain *C. globosum* W7; N2-N6, the *CgligD* gene deletant named *CgligD*-N2–*CgligD*-N6.

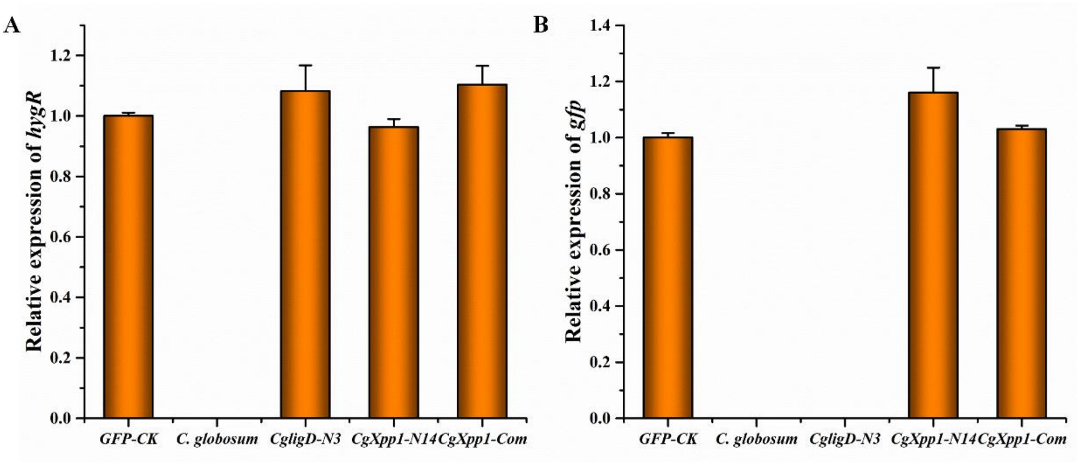

Figure S5. Relative expressions of hygromycin resistance gene (*hygR*) and green fluorescent reporter gene (*gfp*) in GFP-CK (parental strain carrying empty vector pBARGPE1-*EGFP*), *C. globosum*, *CgligD*-N3 (*CgligD* deletion mutant), *CgXpp1*-N14 (*CgXpp1* disruption mutant) and *CgXpp1*-Com (*CgXpp1* complemented derivative) at 9 days of incubation in PDA medium at 28 °C. Data were averaged using triplicate measurements.

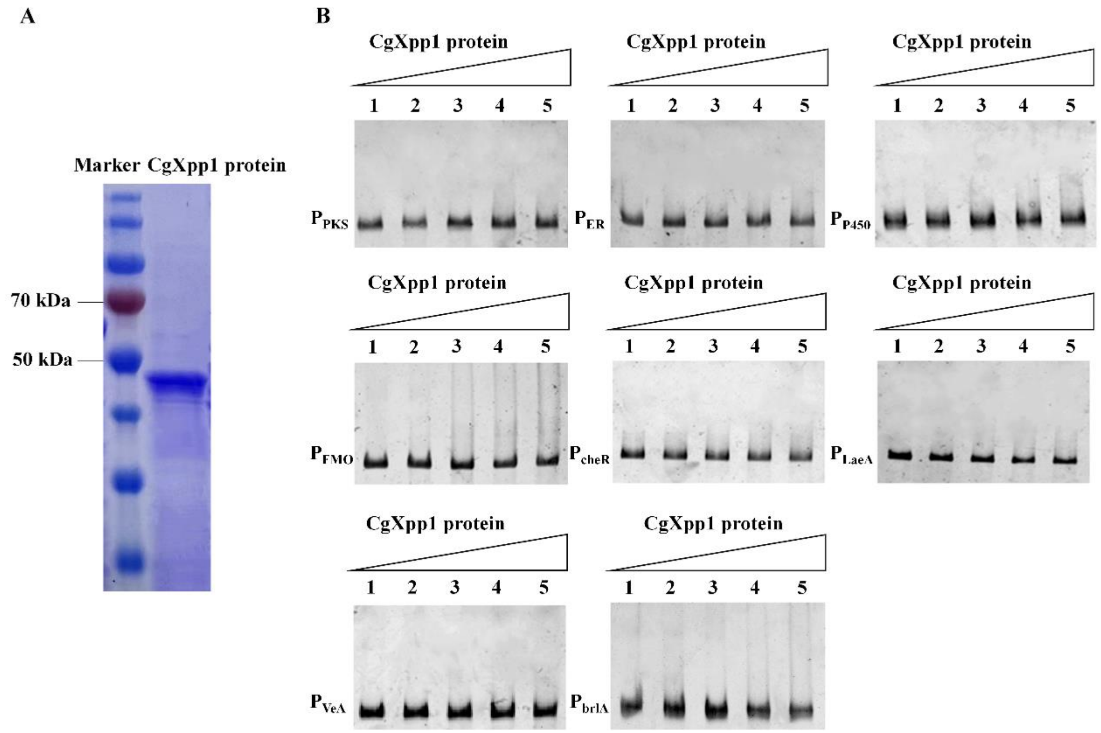

Figure S6. EMSAs for detecting the binding ability of purified CgXpp1 protein to the promoter regions of *CgPKS*, *CgER*, *CgP450*, *CgFMO*, *CgcheR*, *CgLaeA*, *CgVeA* and *brlA*. (A) Validation of the purified CgXpp1 protein by SDS-PAGE; (B) EMSA of the CgXpp1 protein and detected gene probes. Lanes 1–5: 0, 0.4, 0.8, 1.2 and 1.6 μg of CgXpp1, respectively.

Table S1. Primers designed for *C. globosum* W7 in this work.

Primer	Sequence
CgXpp1-ALF	5'- GCAGATATCCATCACACTG**GCGGCCGC**TGCAAGCAGACCGTTGAGCTCC -3' **(NotI)**
CgXpp1-ALR	5'- TGCGTCCGTCCGTCTCTCCGCATGCGGCTGTGAGTCCGGTAAGGGGG-3'
EGFP-F	5'- GCATGCGGAGAGACGGACGGA -3'
EGFP-R	5'- GCAAGTAACTGGGTGGCGAGATGCGTTACTTGTACAGCTCGTCCATGCCG -3'
CgXpp1-ARF	5'- CGCATCTCGCCACCCAGTTACTTGCTA -3'
CgXpp1-ARR	5'-AGGTGTAAACCTTAAACTGCCG**TACGTA**CTCCAACTCCTCCCCACAAACATAA -3' **(SnaBI)**
BlpR-F	5'- ACAGGATGAGGATCGTTTCGCTCGACAGAAGATGATATTGAAGGA -3'
BlpR-R	5'- GAAATTGTAAGCGTTAATAATTCAGATCTCGGTGACGGGC -3'
pBlunt-F	5'- ATTATTAACGCTTACAATTTCCTGATGCGGTATT -3'
pBlunt-R	5'- GCGAAACGATCCTCATCCTGTCTCTTG -3'
p*Kan*-TF	5'- AAAACGCAAGCGCAAAGAGAA -3'
p*Kan*-TR	5'- TTAGAAAAATAAACAAATAGGGGTT -3'
CgXpp1-ComF	5'- AATTCAGGCCTGAATTCTGCA**GATATC**ATGTCCCATGACGTCTCTTCAA -3' **(EcoRV)**
CgXpp1-ComR	5'- CTAGATGCATGCTCGA**GCGGCCGC**CTAACAGAACCCTCTTGGATCGAAAA -3' **(NotI)**
TXF1	5'- CAATACGCAAACCGCCTCTC -3'
TXR1	5'- GACGGCAGTTGAAATGGAATAT -3'
TXF2	5'- TTTTGTTCTTGGTTTGGCTTTG -3'
TXR2	5'- TCAGAGCTTGATCCCCTGCG -3'
CgligD-PTF1	5'- GGTATGTCACCCTTCTTTGGC -3'
CgligD-PTR1	5'- GTTCCTGTCTGCTAATAAGAGTCA -3'
CgligD-PTF2	5'- CCATTCGGACCGCAAGGA -3'
CgligD-PTR2	5'- TGGTGTAGAGCTGGGTGAGGA -3'
CgligD-PTF3	5'- CTCACATTCACAGCCCACCAG -3'
CgligD-PTR3	5'- GCCATACGCCTTTTGACACC -3'
CgXpp1-PTF1	5'- TCAACTTACTTACGGCTCCCCCA -3'
CgXpp1-PTR1	5'- GAGGGGCACACCAGCCTTTC -3'
CgXpp1-PTF2	5'- CCGACCACTACCAGCAGAACAC -3'
CgXpp1-PTR2	5'- TCCGCAACCCGCATCTACAC -3'
CgXpp1-PTF3	5'- ACGATTCATGATGATTAGGCAG -3'
CgXpp1-PTR3	5'- GAATCCAACTTCCTCTACCTAA -3'
CgXpp1-RTF1	5'- ACAATGATGACAGCCTTGCCAG -3'
CgXpp1-RTR1	5'- CGCACTGTCGTCAACTATTTCG -3'
CgVeA-RTF	5'- AGAGCCTGTGGTTCTGGTCCG -3'
CgVeA-RTR	5'- AAAAGTGATGTCCTTCTCCTGCG -3'
CgLaeA-RTF	5'- CCCTCCACAACCAGGAAACTC -3'
CgLaeA-RTR	5'- CGCAAGGAACCGTCATCACTAC -3'

CgCheR-RTF	5'- GTCCGACTGCCTCTACACCACG -3'
CgCheR-RTR	5'- CGATGAACTCGCTACCGCTGA -3'
actin-F	5'-TCATCGACAATGGCTCCGGTATG-3'
actin-R	5'-GCTCGTTGTAGAAGGTGTGATGC-3'
RT-*brlA*-F	5'- CGATGACCGTCCATACACC -3'
RT-*brlA*-R	5'- GCTGAGAGCGGCAGAAGG -3'
RT-ER-F1	5'-GTCTTCACCTCGCCAAAGGGATC-3'
RT-ER-R1	5'-ATGGTTAAACTAACGGGGCATAG-3'
RT-FMO-F1	5'-CGGAAATGCCACGAAGACA-3'
RT-FMO-R1	5'-GGACCAGATGATGGACGATG-3'
RT-PKS-F1	5'-AGGTTCCTCTCGCCATTGCT-3'
RT-PKS-R1	5'-GGCATAGTGATACCTTGCGTTCT-3'
RT-P450-F1	5'-CCAGGTTGGCAAACTTGAA-3'
RT-P450-R1	5'-CAGCCATTTGGTCCTCTATCG-3'
P*CgER*-F	5'-GCTGAAGCGGAGGGAGATT-3'
P*CgER*-R	5'-GCTGTGCCATCCACCGTAG-3'
P*CgPKS*-F	5'-GGCTTGAATCCGCTGTTG-3'
P*CgPKS*-R	5'-TGGAACCTAAAATGAGCG-3'
P*CgP450*-F	5'-AGGCTAAGGTCTTGTTTCG-3'
P*CgP450*-R	5'-GGGAGTGGGGACTGGGTAA-3'
P*CgLaeA*-F	5'-TTGAATCCGTTTCCTGGTCT-3'
P*CgLaeA*-R	5'-TGCGTTTTTTTGCGGTTTTG-3'
P*CgcheR*-F	5'-GTAGAATGGGGTTTAG-3'
P*CgcheR*-R	5'-TCTTTAGACTCCTTACAT-3'
P*CgFMO*-F	5'-CTGGTCTGCTGCGTTGTTG-3'
P*CgFMO*-R	5'-GCCTTGGACTGCTTCGGGA-3'
P*CgVeA*-F	5'-CTCTCCATCAACCAACCTTCATC-3'
P*CgVeA*-R	5'-TGGTCTCTCTGGAGGTGATGGAA-3'
P*blrA*-F	5'-GATTTGGTGCTTGGTACCTTTTCC-3'
P*blrA*-R	5'-AGAGGCAACAACCGAAGAGGAC-3'

Table S2. Metabolites identified that were differentially abundant between *C. globosum* W7 and *CgXpp1*-N14 in the positive ion mode, based on fold $P < 0.05$.

ID	Metabolite identity	log2FC	Pvalue	VIP	Trend	KEGG_pathway_annotation
pos_10474	Thymidine 5'-triphosphate	-11.13423386	0.000164179	1.5687651	down	Pyrimidine metabolism(ko00240)
pos_1060	Gamma-Aminobutyric acid	-13.28867392	0.020709009	1.1940091	down	beta-Alanine metabolism(ko00410); Nicotinate and nicotinamide metabolism(ko00760); Alanine, aspartate and glutamate metabolism(ko00250); Butanoate metabolism(ko00650); Arginine and proline metabolism(ko00330)
pos_1101	Leu Gly Gly	-2.372294194	0.001852061	1.4361394	down	-
pos_11024	trans-delta2, cis-delta4-decadienoyl-CoA	-10.72009982	0.020611046	1.1664208	down	-
pos_11313	N-Carbamoyl-2-amino-2-(4-hydroxyphenyl)acetic acid	12.23586714	0.003395129	1.4284124	up	-
pos_11334	alpha-D-glucosamine 1-phosphate	25.33760871	0.044794898	1.0805267	up	Amino sugar and nucleotide sugar metabolism (ko00520)
pos_11795	Isoleucyl-Isoleucine	-14.99020964	0.000284469	1.5301012	down	-
pos_13050	Phytosphingosine 1-phosphate	23.89639203	0.032245725	1.1418331	up	-
pos_1329	UDP-3-O-(3-hydroxymyristoyl)-N-acetylglucosamine	-13.01163662	0.002382432	1.4609525	down	-

pos_1335	3-Oxodecanoyl-CoA	-13.48854942	0.011739871	1.2203372	down	Fatty acid elongation(ko00062); Fatty acid degradation(ko00071); Fatty acid metabolism(ko01212)
pos_14147	Arachidonoyl dopamine	-12.5842479	0.002325213	1.4494829	down	-
pos_14294	alpha-Linolenic acid	-5.597833544	0.000870852	1.5233314	down	Biosynthesis of unsaturated fatty acids(ko01040); alpha-Linolenic acid metabolism(ko00592)
pos_14357	Stearic acid	-3.642417613	5.38E-05	1.5831907	down	Biosynthesis of unsaturated fatty acids(ko01040); Fatty acid biosynthesis(ko00061)
pos_14473	PI(14:0/16:0)	-9.604069628	0.005109642	1.3879619	down	-
pos_1471	4-Amino-5-hydroxymethyl-2-methylpyrimidine	-18.69520407	9.03E-05	1.6215664	down	Thiamine metabolism(ko00730); ABC transporters(ko02010)
pos_1488	ADP-ribose	-12.44685826	2.27E-06	1.6356617	down	Purine metabolism(ko00230)
pos_15001	PS(19:0/17:0)	-11.14512763	0.003522672	1.370159	down	-
pos_1523	Syringaldehyde	24.81833905	0.013341078	1.2919916	up	-
pos_1556	D-erythro-Sphingosine-1-phosphate	-16.43535231	0.001027893	1.5122714	down	-
pos_15964	PC(24:1(15Z)/16:1(9Z))	-7.114652883	0.029182533	1.1384104	down	Linoleic acid metabolism(ko00591); alpha-Linolenic acid metabolism(ko00592); Glycerophospholipid metabolism(ko00564); Arachidonic acid metabolism(ko00590)
pos_16531	PG(16:1(9Z)/16:0)	-26.98075002	0.022653833	1.1812559	down	-
pos_1879	Deoxyguanosine	-19.28554165	0.014286673	1.2713238	down	Purine metabolism(ko00230)
pos_19465	PC(o-20:0/18:3(9Z,12Z,15Z))	-7.957025211	2.94E-07	1.6471397	down	-

pos_2059	Peonidin 3-O-(coumaroylglucoside)	-16.42131168	0.000511938	1.5681512	down	-
pos_3022	Coenzyme Q10	12.82687033	0.026239719	1.1993753	up	-
pos_3080	Ornithine	-16.1337597	0.00373355	1.4325978	down	D-Arginine and D-ornithine metabolism(ko00472)
pos_3363	Sphinganine 1-phosphate	-8.054890942	0.00062717	1.5517242	down	Sphingolipid metabolism(ko00600)
pos_5245	isoleucine tetrazole	-18.93729924	0.008559165	1.3320553	down	-
pos_5995	beta-D-Fructose 1,6-bisphosphate	18.29135865	0.038619056	1.0991089	up	Pentose phosphate pathway(ko00030); Biosynthesis of amino acids(ko01230); Fructose and mannose metabolism(ko00051); Carbon metabolism(ko01200); Glycolysis/Gluconeogenesis(ko00010)
pos_6261	Diaminopimelic acid	-7.275815377	0.000143543	1.585038	down	Biosynthesis of amino acids(ko01230); Lysine biosynthesis(ko00300)
pos_6262	Orotate	-13.90198905	0.002691629	1.4186936	down	Pyrimidine metabolism(ko00240)
pos_66	Triethanolamine	-7.101219122	4.10E-06	1.6027386	down	Glycerophospholipid metabolism(ko00564)
pos_7051	2-dehydro-D-gluconate	13.42904738	0.015717827	1.2782688	up	Pentose phosphate pathway(ko00030)
pos_711	D-Ribulose 5-phosphate	-1.664688671	0.007843919	1.309667	down	Riboflavin metabolism(ko00740); Methane metabolism(ko00680); Biosynthesis of amino acids(ko01230);
pos_7325	Dethiobiotin	-14.81363637	0.005477542	1.348116	down	Biotin metabolism(ko00780)

pos_743	AICAR	-7.942117991	2.01E-05	1.5640045	down	Purine metabolism(ko00230); Histidine metabolism(ko00340)
pos_8165	Leucyl-Isoleucine	-10.84923417	0.007264035	1.3007764	down	-
pos_9080	dATP	-14.93449959	0.01024357	1.2656835	down	Purine metabolism (ko00230)
pos_12491	Kanzonol W	5.083316532	0.034980134	1.1479766	up	-
pos_12483	Isodictamnine	2.607669063	0.035526969	1.1417375	up	-
pos_12623	cephalexin	1.79147002	0.024021274	1.1908015	up	-
pos_12981	Pantetheine 4'-phosphate	23.37940416	0.042838923	1.0923054	up	Carbapenem biosynthesis(ko00332); Pantothenate and CoA biosynthesis(ko00770)
pos_14315	PS(19:0/18:1(11Z))	-8.977154946	6.65E-05	1.6261843	down	-
pos_151	Pyridoxamine	-3.009979397	2.38E-06	1.6150199	down	Vitamin B6 metabolism(ko00750)

Table S3. Metabolites identified that were differentially abundant between *C. globosum* W7 and *CgXpp1*-N14 in the negative ion mode, based on fold $P < 0.05$.

ID	name	log2FC	Pvalue	VIP	Trend	KEGG
neg_10062	3-dehydroquinate	-13.37343439	0.029913288	1.043651166	down	Biosynthesis of amino acids(ko01230)
neg_10071	L-erythro-tetrahydrobiopterin	-14.78238948	0.000108622	1.491899232	down	Folate biosynthesis(ko00790); Metabolic pathways(ko01100)
neg_10088	Alfuzosin	14.30754268	0.028879148	1.159931132	up	-
neg_10201	Aclarubicin	17.23339309	0.02820355	1.176734898	up	-
neg_10296	Tetrahydrobiopterin	-12.23798691	0.000241059	1.500244583	down	Metabolic pathways(ko01100); Folate biosynthesis(ko00790)
neg_10329	Fluconazole	10.84352267	0.034197768	1.134783737	up	-
neg_10447	Ginkgolide J	14.97121802	0.021110074	1.153519273	up	-
neg_10469	Pyrazinamide	21.26914237	0.029021777	1.109206374	up	-
neg_10556	Ureidoisobutyric acid	16.23865138	0.010266139	1.269250335	up	-
neg_10871	ubiquinone-0	-17.3003593	0.013301034	1.223316759	down	-
neg_11401	3-Carboxy-2,3,4,9-tetrahydro-1H-pyrido[3,4-b]indole-1-propanoic acid	-18.08313867	0.000447415	1.492440652	down	-

neg_11763	Agmatine	-5.001250506	0.014493821	1.144251727	down	Metabolic pathways(ko01100); Arginine and proline metabolism(ko00330)
neg_12543	2-aminogalactopyranose	16.71476365	0.024898431	1.181086051	up	-
neg_12812	His Gln Asn Glu	-3.432330967	1.71E-06	1.54422876	down	-
neg_13574	S-Adenosylmethionine	-3.109457794	0.000514586	1.419061938	down	Monobactam biosynthesis(ko00261); Biosynthesis of amino acids(ko01230)
neg_13615	PS(14:1(9Z)/16:0)	-6.508461852	0.010807343	1.199500777	down	-
neg_13729	PG(16:0/18:1(11Z))	-5.222676366	0.007158976	1.196056795	down	-
neg_13831	Mefenamic acid	-17.56825452	0.000278413	1.520582704	down	-
neg_13900	hexadec-2-enoyl-CoA	13.53803964	0.028751036	1.107196147	up	-
neg_14191	Arginyl-Methionine	-17.83834032	9.88E-06	1.57661586	down	-
neg_14221	Thr Ile Ser Glu	-11.48106467	0.001101947	1.433904779	down	-
neg_14261	Tricosanoic acid	-18.17157446	0.042085592	1.006254273	down	-
neg_1666	Adenosine 3'-monophosphate	-0.916673661	0.032578306	1.043213254	down	Purine metabolism(ko00230); Metabolic pathways(ko01100)
neg_1845	2-Hydroxycinnamic acid	-2.308390373	9.19E-05	1.509469961	down	Phenylalanine metabolism(ko00360)

neg_1850	7,8-dihydroneopterin 3'-phosphate	-2.848106012	4.12E-05	1.525257887	down	Folate biosynthesis(ko00790)
neg_1858	Cyclic GMP	-1.193961723	0.003255237	1.317660351	down	Purine metabolism(ko00230)
neg_1869	3'-AMP	-2.875242595	1.45E-05	1.520874958	down	Purine metabolism(ko00230)
neg_1897	Ascorbic acid	-11.0855945	0.024747492	1.116019191	down	Glutathione metabolism(ko00480); Ascorbate and aldarate
neg_2004	O-Phosphoethanolamine	-2.689966496	7.58E-07	1.545758676	down	Ubiquinone and other terpenoid-quinone biosynthesis(ko00130)
neg_2351	E-Linalool oxide	-10.02333077	0.004269877	1.345978622	down	-
neg_2531	GMP-lysine	-11.81656003	0.000260553	1.415914991	down	-
neg_2532	Histidinyl-Cysteine	-11.30134226	0.000135149	1.495158879	down	-
neg_2555	Deoxyribose	-8.53068332	3.16E-06	1.537288418	down	Pentose phosphate pathway(ko00030)
neg_2558	Arginyl-Tyrosine	-15.57281123	0.008506452	1.270447332	down	-
neg_2564	Maltotriose	-14.44129146	0.001903019	1.408322775	down	ABC transporters(ko02010)
neg_2615	Cytidine 5'-monophosphate (CMP)	-18.35439445	0.003588193	1.364760025	down	-
neg_2676	Octanoyl-CoA	-10.98153984	0.036984331	1.02188744	down	Lipoic acid metabolism(ko00785); Fatty acid metabolism(ko01212)
neg_2723	Malvidin 3-O-(acetylglucoside)	-11.68701603	0.009780569	1.254724788	down	-

neg_3822	PS(19:0/16:0)	-10.59380226	0.001481527	1.422296958	down	-
neg_4169	PI(16:1(9Z)/18:1(9Z))	-11.63209157	0.000614401	1.468455794	down	-
neg_4172	PI(18:1(9Z)/18:1(9Z))	-14.23931772	0.000168119	1.520512911	down	-
neg_4354	PG(16:0/19:0)	-9.489451811	0.026889955	1.148301973	down	-
neg_5458	4-acetamidobutanal	-11.20682122	0.000760727	1.460599905	down	Arginine and proline metabolism(ko00330);
neg_5553	Anserine	-5.018091507	0.024437981	1.074309987	down	Histidine metabolism(ko00340); beta-Alanine metabolism(ko00410)
neg_6061	Thr Ile Gln Asp	-6.159694054	0.00171791	1.298149322	down	-
neg_6611	L-Fucose	-3.259300346	0.004296607	1.341780358	down	Amino sugar and nucleotide sugar metabolism(ko00520);
neg_671	O-Phosphorylethanolamine	-1.393313702	0.000491963	1.3970652	down	Sphingolipid metabolism(ko00600)
neg_8304	NMNH	20.93609062	0.015608154	1.267944225	up	Biosynthesis of secondary metabolites(ko01110); Aflatoxin
neg_9216	Sterigmatocystin	1.268193113	0.004874468	1.309134317	up	Biosynthesis of secondary metabolites(ko01110); Aflatoxin
neg_9342	Manumycin A	20.63651094	0.013054371	1.175089455	up	-
neg_9385	3-(2,3-Dihydroxyphenyl) propanoate	-12.52020538	0.009917368	1.272878889	down	Phenylalanine metabolism(ko00360)
neg_9452	formycin B	1.449119826	0.02684409	1.105033007	up	-

neg_9703	UDPglucose	-10.66644655	0.003785958	1.245492312	down	Pyrimidine metabolism(ko00240); Ascorbate and aldarate
neg_9870	Zafirlukast	8.188162101	0.038520837	1.055172536	up	-
